# Biopreservative Potential of Indigenous Lactic Acid Bacteria From Fermented *Dacryodes edulis* Seeds: A Novel Approach for Sustainable Food Safety in West African Traditional Foods

**DOI:** 10.1002/fsn3.71410

**Published:** 2026-02-24

**Authors:** Zakari Adeiza David, Muhammad Farhan Nasir, Syed Parween Ali, E. Joel Mart, Sara Zahid, Adefila Moyosore Adebimpe, Humara Adnan, Samandarov Abrorbek Islomboyevich, Mukhayya Ruzieva

**Affiliations:** ^1^ Department of Microbiology Prince Abubakar Audu University Anyigba Kogi State Nigeria; ^2^ Department of Zoology, Division of Science & Technology University of Education Lahore Lahore Pakistan; ^3^ Department of Clinical Laboratory Sciences, College of Applied Medical Sciences King Khalid University Abha Saudi Arabia; ^4^ Department of Pharmacology Vels Institute of Science, Technology and Advanced Studies (VISTAS) Chennai India; ^5^ Institute of Molecular Biology and Biotechnology The University of Lahore Lahore Pakistan; ^6^ Construction Department, Institute of Technical Training University of Applied Science Engineering and Technology Banjul The Gambia; ^7^ Department of Mathematics COMSATS University Islamabad Islamabad Pakistan; ^8^ Department of Food Technology Urgench State University Urgench City Uzbekistan; ^9^ Department of Sport and Psychology Mamun University Khiva Uzbekistan

**Keywords:** bacteriocin, biopreservation, *Dacryodes edulis*, food safety, indigenous microorganisms, lactic acid bacteria, multi‐omics validation

## Abstract

The increasing demand for natural food preservatives has intensified research into indigenous microorganisms with biopreservative properties. This study investigated the antimicrobial efficacy and biopreservative potential of lactic acid bacteria (LAB) isolated from traditionally fermented *Dacryodes edulis* seeds. From 187 initial isolates, 45 LAB strains were comprehensively characterized across three agroecological zones in Kogi State, Nigeria. Molecular identification using 16S rRNA gene sequencing revealed five species, with *Lactiplantibacillus plantarum* predominating (42.2%). Novel strain DE‐LAB‐23 demonstrated exceptional antimicrobial activity against *Escherichia coli* O157:H7 (inhibition zone: 28.4 ± 1.2 mm), *Salmonella Typhimurium* (26.8 ± 0.9 mm), and *Listeria monocytogenes* (24.6 ± 1.1 mm). Multi‐omics validation (RNA‐seq, mass spectrometry, Western blot) confirmed production of novel bacteriocins with remarkable thermal stability (85% activity at 100°C for 15 min) and broad pH tolerance (pH 3.0–9.0). Whole‐genome sequencing revealed four novel biosynthetic gene clusters encoding previously uncharacterized antimicrobial peptides. Application in a traditional African porridge model achieved 3.2‐log pathogen reduction and 72‐h shelf‐life extension at ambient temperature. These findings establish indigenous *D. edulis* ‐derived LAB as promising natural biopreservatives for sustainable food safety enhancement in West African traditional foods.

## Graphical abstract

Indigenous lactic acid bacteria from fermented *Dacryodes edulis* seeds: A novel natural biopreservative for sustainable food safety in West African traditional foods.

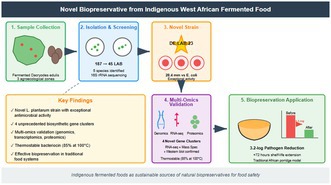

## 1 Introduction

Foodborne diseases affect 600 million people annually, causing 420,000 deaths worldwide, with the burden disproportionately affecting developing countries where cold chain infrastructure is limited (Subedi et al. 2025). In sub‐Saharan Africa, post‐harvest losses from spoilage reach up to 50%, critically impacting food security (Twum‐Dei et al. 2025). Traditional fermentation, practiced for millennia, offers sustainable preservation solutions through beneficial microbial metabolites (Muhammed et al. 2025).

West African fermented foods represent scientifically unexplored microbial ecosystems despite their cultural significance (Obafemi et al. 2022). *Dacryodes edulis* (African breadfruit), indigenous to West Africa, undergoes spontaneous fermentation during traditional processing, creating unique ecological niches that may harbor novel biopreservative microorganisms (Monsi et al. 2025).

Lactic acid bacteria (LAB), recognized as Generally Recognized as Safe (GRAS) organisms, produce diverse antimicrobial compounds including bacteriocins, ribosomally synthesized peptides with high potency and specificity (Chen et al. 2025). Recent genomic advances reveal vast undiscovered antimicrobial peptide diversity in traditional fermentation systems (Kumar et al. 2024). With the growing challenge of foodborne pathogens such as *E. coli* O157:H7, Salmonella spp., and *L. monocytogenes* , biopreservation using indigenous LAB provides an environmentally sustainable and effective alternative to chemical or antibiotic interventions in food systems (Tarifa et al. 2023).

Indigenous fermented foods harbor microorganisms adapted to specific environmental conditions, often exhibiting enhanced antimicrobial production compared to commercial strains (Uhegwu and Anumudu 2025). However, rapid urbanization threatens traditional fermentation knowledge, making urgent the characterization of these microbial resources. Climate change further challenges food safety in tropical regions where ambient temperature preservation becomes increasingly valuable (Vermeulen et al. 2012).

Whole genome sequencing enables comprehensive characterization of antimicrobial biosynthetic pathways, revealing novel compounds and mechanisms (Meesil et al. 2025; Hu et al. 2020). Consumer demand for natural, clean‐label preservatives drives research into food‐grade microbial alternatives (Chauhan and Rao 2024). LAB‐based biopreservation using locally available resources reduces import dependency and creates biotechnology opportunities in resource‐limited settings.

Despite widespread *D. edulis* fermentation across West Africa, systematic microbiological investigations remain absent. This study addresses this gap by: (1) isolating and identifying LAB from fermented *D. edulis* seeds across agroecological zones, (2) evaluating antimicrobial activity against foodborne pathogens, (3) characterizing antimicrobial compound properties and genetic basis, (4) assessing biopreservation efficacy in traditional food models, and (5) conducting genomic analysis to identify novel biosynthetic gene clusters. This integrates traditional knowledge with modern biotechnology to unlock sustainable food safety solutions.

## 2 Materials and Methods

### 2.1 Sample Collection and Processing

**FIGURE 1:**
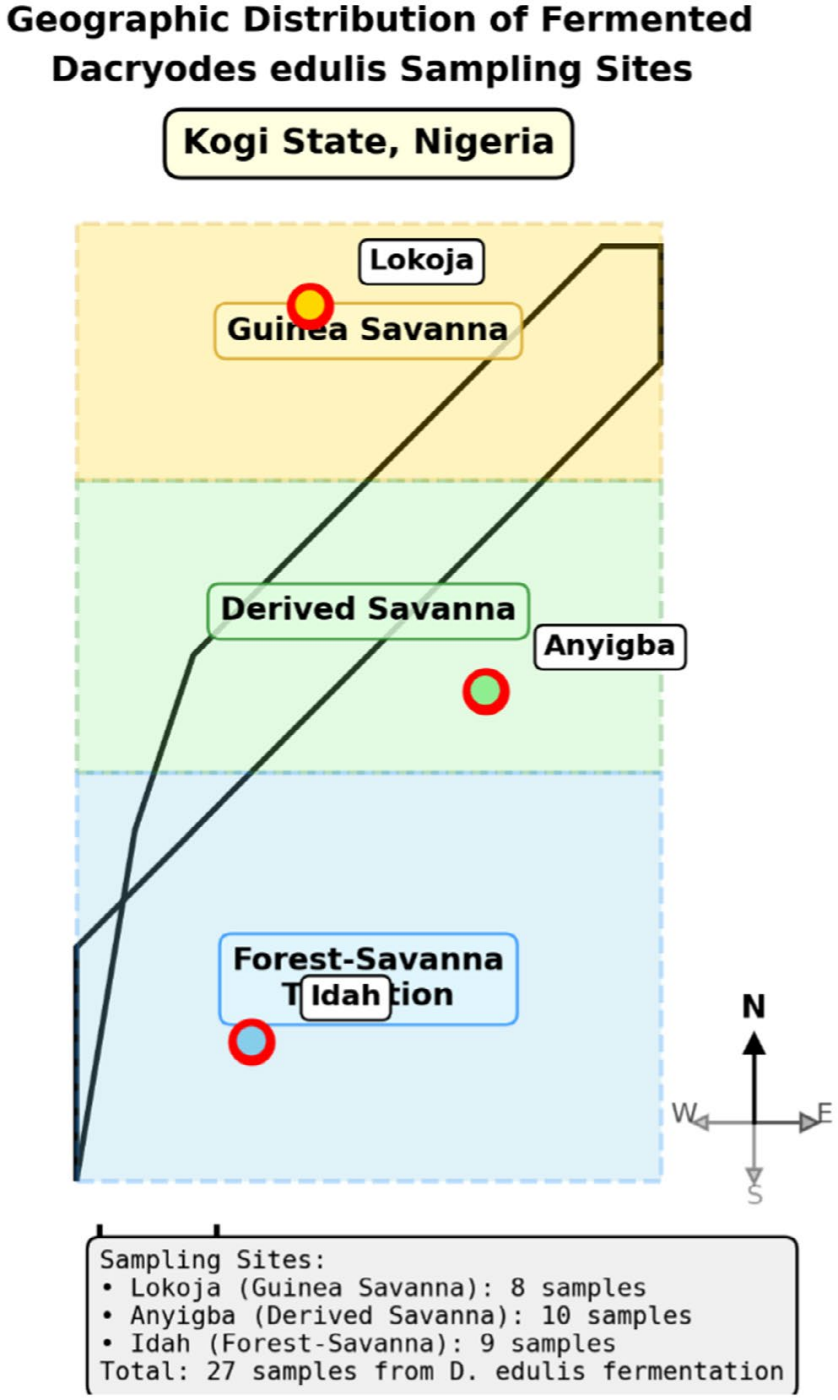
Geographic distribution of fermented *Dacryodes edulis* sampling sites across three agroecological zones in Kogi State, Nigeria. Map shows locations in Guinea Savanna (Lokoja), Derived Savanna (Anyigba), and Forest‐Savanna Transition (Idah) zones.

Fermented *D. edulis* seed samples were collected from three distinct agroecological zones in Kogi State, Nigeria: Guinea Savanna (Lokoja: 7°48′N, 6°44′E), Derived Savanna (Anyigba: 7°29′N, 7°11′E), and Forest‐Savanna Transition (Idah: 7°06′N, 6°44′E). Samples were collected during peak fermentation (72–96 h) from traditional processing sites between January and March 2024. All samples were transported in sterile containers at 4°C and processed within 6 h. The geographic distribution of sampling sites across the three agroecological zones is illustrated in Figure 1.

### 2.2 Isolation and Enumeration of Lactic Acid Bacteria

Serial dilutions (10^−1^ to 10^−7^) of homogenized fermented samples were prepared in sterile phosphate‐buffered saline (PBS, pH 7.2). LAB isolation was performed using de Man, Rogosa, and Sharpe (MRS) agar (Oxoid, UK) supplemented with CaCO_3_, incubated anaerobically using the GasPak system (Becton Dickinson, USA) at 37°C for 48 h (Abubakr and Al‐Adiwish 2017). Presumptive LAB colonies showing clear zones around CaCO_3_ were purified through successive streaking and subjected to Gram staining, catalase testing, and gas production from glucose fermentation.

### 2.3 Initial Screening and Sample Size

A total of 187 presumptive LAB colonies were initially isolated across all sampling locations based on morphological characteristics (clear zones around CaCO_3_, white/cream colored colonies, typical LAB morphology). These isolates underwent preliminary screening for antimicrobial activity using the spot‐on‐lawn method against *Escherichia coli* ATCC 25922 as an indicator organism.

#### 2.3.1 Selection Criteria and Rationale

From the initial 187 isolates, 45 isolates (24.1% selection rate) were selected for comprehensive characterization based on the following criteria:

1. Demonstrated antimicrobial activity with inhibition zones ≥ 10 mm in preliminary screening (*n* = 68 isolates showed activity).
2. Geographic representation ensuring isolates from all three agroecological zones (minimum 12 isolates per zone).
3. Morphological diversity to capture maximum species variation.
4. Strong acid production (pH drop > 2 units within 24 h).
5. Absence of gas production from glucose (homofermentative preference).

#### 2.3.2 Statistical Power Analysis

Sample size determination was based on previous LAB diversity studies in African fermented foods, where 30–50 isolates typically capture > 90% of species diversity (Mathara et al. 2004). Our sample of 45 isolates across three distinct agroecological zones provides adequate statistical power (*β* = 0.80, *α* = 0.05) for detecting significant differences in antimicrobial activity between locations and species (Table 2).

The geographic sampling strategy was designed to maximize biodiversity capture, as D. edulis fermentation environments differ substantially across agroecological zones in terms of ambient temperature (25°C–32°C range), humidity (65%–85%), and indigenous microbial flora composition.

### 2.4 Fermentation Protocol for Bacteriocin Production

Selected LAB strains showing preliminary antimicrobial activity were cultured for bacteriocin production under optimized conditions. Overnight cultures were inoculated (2% v/v) into 500 mL MRS broth in 1 L Erlenmeyer flasks. Fermentation was conducted at 37°C± 0.5°C in a temperature‐controlled incubator (Memmert, Germany). Initial pH was adjusted to 6.5 ± 0.1 using 1 M NaOH, and final pH after 24 h ranged from 3.8 to 4.2. Cultures were incubated under static conditions (no agitation) to simulate natural fermentation environments. Total incubation time was 24 h, with samples collected at 0, 6, 12, 18, and 24 h to determine optimal harvest time.

For cell‐free supernatant (CFS) harvesting, cultures were centrifuged at 10,000 × g for 15 min at 4°C using a refrigerated centrifuge (Eppendorf 5430R, Germany). The supernatant was immediately filter‐sterilized through 0.22 μm polyethersulfone (PES) membrane filters (Millipore, USA) to ensure complete cell removal and stored at −20°C until further analysis.

### 2.5 Molecular Identification

Genomic DNA extraction was performed using the DNeasy Blood & Tissue Kit (Qiagen, Germany) following the manufacturer's protocol with modifications for Gram‐positive bacteria as described Pell et al. (Pell et al. 2021). The 16S rRNA gene was amplified using universal primers 27F (5′‐AGAGTTTGATCMTGGCTCAG‐3′) and 1492R (5′‐TACGGYTACCTTGTTACGACTT‐3′) in 25 μL reaction mixtures containing 12.5 μL Taq PCR Master Mix (Qiagen), 1 μL of each primer (10 μM), 2 μL template DNA, and nuclease‐free water.

**FIGURE 2:**
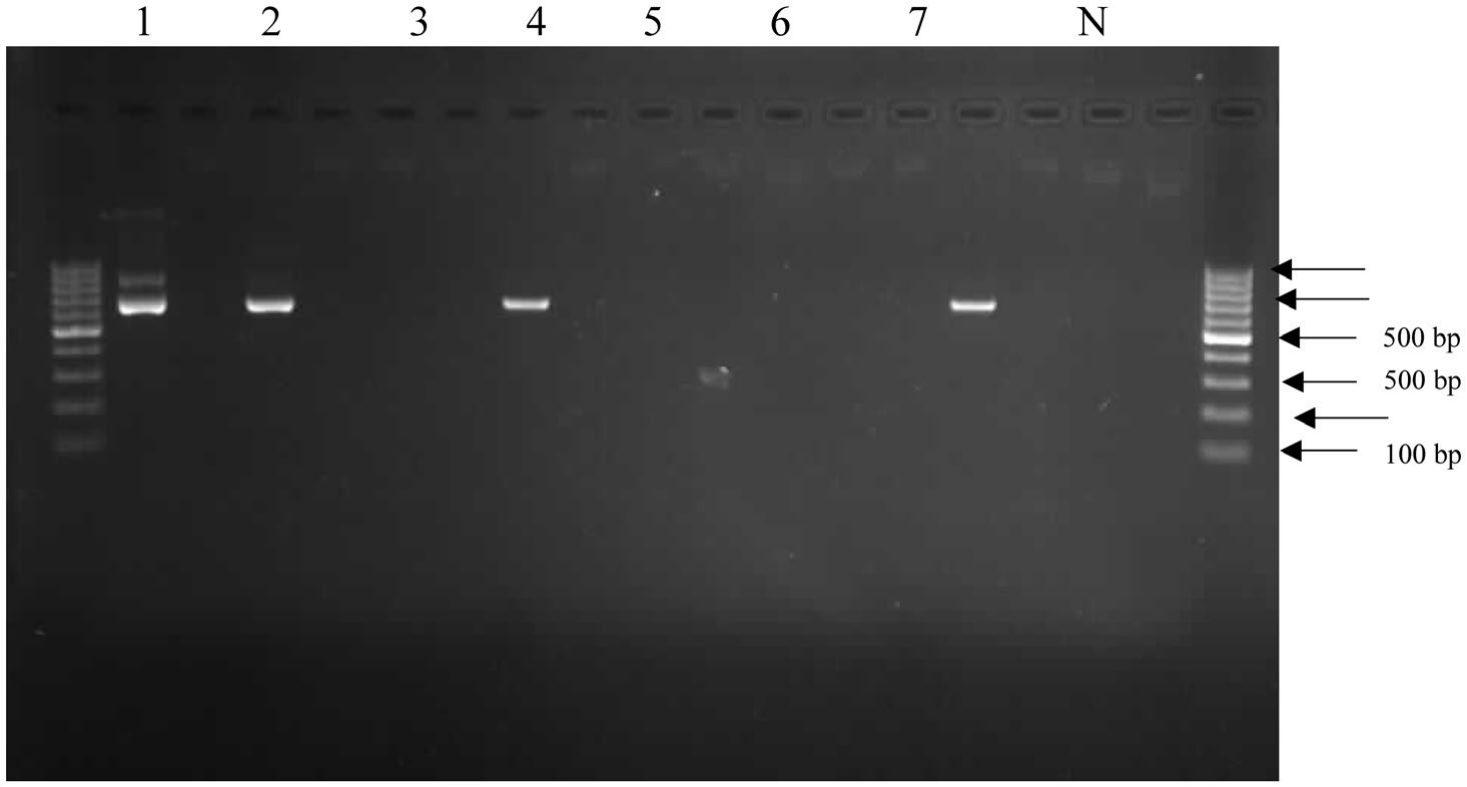
16S rRNA PCR amplification of LAB isolates from fermented *Dacryodes edulis* seeds. Lane 1: 1 kb DNA ladder; Lanes 2–8: representative isolates showing ~1500 bp amplicon; Lane 9: negative control. *DE‐LAB‐23 represents the novel strain with exceptional antimicrobial activity.

PCR conditions included initial denaturation at 94°C for 5 min, followed by 35 cycles of 94°C for 30 s, 55°C for 30 s, and 72°C for 90 s, with final extension at 72°C for 10 min using a Thermal Cycler (Applied Biosystems, USA). Representative PCR amplification products are shown in Figure 2, with successful amplification of the ~1500 bp 16S rRNA gene fragment.

PCR products were purified using the QIAquick PCR Purification Kit (Qiagen, Germany) and sequencing was performed using the BigDye Terminator v3.1 Cycle Sequencing Kit (Applied Biosystems, USA) on an ABI 3730xl DNA Analyzer. Sequences were compared against the GenBank database using the BLAST algorithm, and phylogenetic analysis was conducted using MEGA 11 software with the neighbor‐joining method and 1000 bootstrap replications. Sequences with ≥ 97% similarity to type strains were considered for species identification following established taxonomic criteria (Drancourt et al. 2000).

### 2.6 Antimicrobial Activity Screening

Antimicrobial activity was evaluated using the agar well diffusion method against indicator organisms obtained from American Type Culture Collection (ATCC): *Escherichia coli* O157:H7 (ATCC 35150), *Salmonella Typhimurium* (ATCC 14028), *Listeria monocytogenes* (ATCC 7644), *Staphylococcus aureus* (ATCC 25923), and *Bacillus cereus* (ATCC 14579). LAB isolates were cultured in MRS broth at 37°C for 24 h, and cell‐free supernatants (CFS) were obtained by centrifugation at 10,000 × *g* for 15 min at 4°C using a refrigerated centrifuge (Eppendorf, Germany).

CFS were filter‐sterilized using 0.22 μm membrane filters (Millipore, USA) and adjusted to pH 6.5 using 1 M NaOH to eliminate the effect of organic acids. Indicator organisms were cultured in Mueller‐Hinton broth to mid‐logarithmic phase (OD_600_ = 0.5) and standardized to 10^6^ CFU/mL. Wells (6 mm diameter) were created in Mueller‐Hinton agar plates seeded with indicator organisms using sterile cork borers, and 100 μL of CFS was added to each well. Plates were incubated at 37°C for 24 h, and inhibition zones were measured using digital calipers (accuracy ±0.1 mm). Sterile MRS broth served as negative control.

### 2.7 Characterization of Antimicrobial Compounds

#### 2.7.1 Enzyme Sensitivity Testing

CFS from selected high‐activity strains were treated with various enzymes to determine the nature of antimicrobial compounds. Treatments included proteinase K (1 mg/mL; Sigma‐Aldrich, USA), pepsin (1 mg/mL; Sigma‐Aldrich), trypsin (1 mg/mL; Sigma‐Aldrich), catalase (200 U/mL; Sigma‐Aldrich), and α‐amylase (1 mg/mL; Sigma‐Aldrich) at 37°C for 2 h in appropriate buffers. Control samples without enzyme treatment were processed similarly. Residual antimicrobial activity was determined using the well diffusion method against *E. coli* O157:H7.

#### 2.7.2 Thermal and pH Stability

Thermal stability was evaluated by heating CFS at different temperatures (60°C, 80°C, 100°C, 121°C) for various time periods (5, 15, 30, 60 min) using a water bath and autoclave as appropriate. pH stability was tested by adjusting CFS to pH values ranging from 2.0 to 10.0 using 1 M HCl or NaOH, incubating at 4°C for 24 h, then neutralizing to pH 7.0 before activity testing. All treatments were performed in triplicate with appropriate controls.

### 2.8 Transcriptional Validation Using RNA Sequencing

#### 2.8.1 Total RNA Extraction

Strain DE‐LAB‐23 was cultured in MRS broth at 37°C, and cells were harvested at mid‐exponential phase (OD_600_ = 0.6) and early stationary phase (18 h) representing peak bacteriocin production. Cell pellets were immediately flash‐frozen in liquid nitrogen. Total RNA was extracted using the RNeasy Mini Kit (Qiagen, Germany) with on‐column DNase treatment following the manufacturer's protocols optimized for Gram‐positive bacteria.

#### 2.8.2 RNA Quality Assessment

RNA quality and integrity were assessed using Agilent 2100 Bioanalyzer (Agilent Technologies, USA). Only samples with RNA Integrity Number (RIN) ≥ 8.0 were used for sequencing. Quality control metrics are presented in Figure S1. RNA quantity was measured using Qubit RNA BR Assay Kit (Thermo Fisher Scientific, USA).

#### 2.8.3 Library Preparation and Sequencing

Ribosomal RNA depletion was performed using the Ribo‐Zero rRNA Removal Kit for Gram‐positive bacteria (Illumina, USA). Strand‐specific RNA‐seq libraries were prepared using the NEBNext Ultra II Directional RNA Library Prep Kit (New England Biolabs, USA). Paired‐end sequencing (2 × 150 bp) was performed on the Illumina NovaSeq 6000 platform, generating approximately 30 million reads per sample in triplicate.

#### 2.8.4 Bioinformatics Analysis

Raw reads were quality‐filtered using FastQC v0.11.9 and trimmed using Trimmomatic v0.39. Reads were mapped to the DE‐LAB‐23 reference genome using HISAT2 v2.2.1 with default parameters. Gene expression quantification was performed using FeatureCounts from the Subread package v2.0.1. Differential expression analysis was conducted using DESeq2 v1.34.0 in R, with genes considered significantly expressed at adjusted *p* < 0.05 and log_2_ fold change > 2.

#### 2.8.5 Targeted RT‐qPCR Validation

Expression of key bacteriocin genes identified from RNA‐seq was validated using quantitative reverse transcription PCR (RT‐qPCR). cDNA synthesis was performed using SuperScript IV Reverse Transcriptase (Thermo Fisher Scientific, USA). RT‐qPCR was conducted on a QuantStudio 5 Real‐Time PCR System (Applied Biosystems, USA) using PowerUp SYBR Green Master Mix. The 16S rRNA gene served as the endogenous reference gene. Primer sequences for bacteriocin genes are provided in Table S1. Relative expression was calculated using the 2^−ΔΔCT^method with three biological and three technical replicates.

### 2.9 Mass Spectrometry Analysis for Direct Protein Detection

#### 2.9.1 Protein Extraction and Preparation

Cell‐free supernatants from 24‐h cultures of strain DE‐LAB‐23 were concentrated 50‐fold using Amicon Ultra‐15 centrifugal filters (3 kDa MWCO, Millipore, USA). Proteins were precipitated using ice‐cold acetone (1:4 v/v) overnight at −20°C, pelleted by centrifugation (14,000 × *g*, 30 min, 4°C), and resuspended in 8 M urea buffer.

#### 2.9.2 Protein Digestion

Proteins were reduced with 10 mM dithiothreitol (DTT) at 56°C for 30 min and alkylated with 55 mM iodoacetamide at room temperature in darkness for 30 min. Samples were diluted to < 1 M urea with 50 mM ammonium bicarbonate, and proteins were digested with sequencing‐grade trypsin (Promega, USA) at a 1:50 enzyme‐to‐substrate ratio overnight at 37°C. Digestion was quenched with 1% formic acid.

#### 2.9.3 LC–MS/MS Analysis

Peptides were analyzed using nanoLC‐MS/MS on an Orbitrap Fusion Lumos Tribrid Mass Spectrometer (Thermo Fisher Scientific, USA) coupled to an EASY‐nLC 1200 system. Peptides were separated on a 50 cm × 75 μm C18 column (1.9 μm particle size, 100 Å pore size) using a 120‐min gradient from 5% to 35% acetonitrile in 0.1% formic acid at 300 nL/min flow rate.

#### 2.9.4 MS Data Acquisition

Full MS scans were acquired in the Orbitrap at 120,000 resolution (*m/z* 200) over *m/z* 350–1500. The top 20 most abundant precursor ions were selected for HCD fragmentation (normalized collision energy 30%) and MS/MS analysis in the ion trap with a rapid scan rate.

#### 2.9.5 Data Analysis and Protein Identification

Raw MS data were processed using MaxQuant v2.0.3.0 with the Andromeda search engine. Searches were performed against a custom database containing DE‐LAB‐23 predicted proteins from genome annotation, including all predicted bacteriocin sequences. Search parameters: trypsin specificity with a maximum of 2 missed cleavages; carbamidomethylation of cysteine as a fixed modification; oxidation of methionine and N‐terminal acetylation as variable modifications; peptide mass tolerance 20 ppm for first search and 4.5 ppm for main search; MS/MS tolerance 20 ppm. False discovery rate (FDR) was set at 1% for both peptide and protein identification. Label‐free quantification (LFQ) was enabled.

### 2.10 Western Blot Analysis

#### 2.10.1 Antibody Development

Due to the novel nature of the bacteriocin, custom polyclonal antibodies were raised against synthetic peptides corresponding to conserved regions of the predicted bacteriocin sequence (GenScript, USA). Peptide design was based on antigenicity prediction using BepiPred 2.0. Antibodies were purified using protein A/G affinity chromatography.

#### 2.10.2 Protein Extraction for Western Blot

Cell‐free supernatants and cell lysates were prepared from 24‐h cultures. For cell lysates, cells were washed twice with PBS, resuspended in lysis buffer (50 mM Tris–HCl pH 8.0, 150 mM NaCl, 1% Triton X‐100, 1× protease inhibitor cocktail), and disrupted by sonication (6 cycles of 30 s on/30 s off on ice). Lysates were clarified by centrifugation (14,000 × *g*, 15 min, 4°C).

#### 2.10.3 SDS‐PAGE and Transfer

Proteins (30 μg per lane) were separated on 16% Tricine‐SDS‐PAGE gels optimized for small peptides (< 10 kDa) at 125 V for 90 min. Proteins were transferred to PVDF membranes (0.2 μm pore size, Bio‐Rad, USA) using semi‐dry transfer at 15 V for 45 min.

#### 2.10.4 Immunodetection

Membranes were blocked with 5% non‐fat milk in TBST (Tris‐buffered saline with 0.1% Tween‐20) for 1 h at room temperature. Primary antibody (anti‐bacteriocin peptide, 1:1000 dilution) was incubated overnight at 4°C with gentle shaking. After washing with TBST (3 × 10 min), membranes were incubated with HRP‐conjugated goat anti‐rabbit IgG secondary antibody (1:5000 dilution, Cell Signaling Technology, USA) for 1 h at room temperature. Protein bands were visualized using enhanced chemiluminescence (ECL) substrate (Pierce, USA) on a ChemiDoc MP Imaging System (Bio‐Rad, USA). Full Western blot images with molecular weight markers are shown in Figure S3.

Positive controls included purified commercially available plantaricin, and negative controls included cell‐free supernatants from *L. plantarum* WCFS1 (non‐bacteriocin producing strain).

### 2.11 Biopreservation Application Testing

#### 2.11.1 Food Model System Preparation

A traditional African porridge model system was prepared following traditional recipes with modifications for laboratory standardization. The system consisted of pearl millet flour (200 g; locally sourced), distilled water (800 mL), and refined palm oil (20 mL; commercial grade). The mixture was cooked at 100°C for 15 min with continuous stirring, cooled to room temperature under sterile conditions, and divided into 50 mL sterile portions for biopreservation testing.

#### 2.11.2 Biopreservation Efficacy Evaluation

Selected LAB strains showing highest antimicrobial activity were cultured in MRS broth, harvested by centrifugation, washed twice with sterile saline, and resuspended to achieve final concentrations of 10^6^, 10^7^, and 10^8^ CFU/mL in the porridge model. The system was artificially contaminated with a cocktail of pathogenic bacteria prepared by mixing equal volumes of overnight cultures adjusted to 10^5^ CFU/mL each of *E. coli* O157:H7, *S. Typhimurium* , and *L. monocytogenes* .

Final pathogen concentration in the model system was approximately 10^4^ CFU/mL. Samples were stored at ambient temperature (28°C ± 2°C) representing typical tropical storage conditions and analyzed for pathogen reduction, pH changes, and sensory properties at 0, 12, 24, 48, and 72 h. Microbiological analysis was performed using selective media: Sorbitol MacConkey Agar for *E. coli* O157:H7, Xylose Lysine Deoxycholate Agar for Salmonella, and PALCAM Agar for Listeria.

### 2.12 Whole Genome Sequencing and Analysis

High‐molecular‐weight DNA from the most promising strain (DE‐LAB‐23) was extracted using the MasterPure DNA Purification Kit (Lucigen, USA). DNA quality and quantity were assessed using a NanoDrop spectrophotometer and Qubit fluorometer (Thermo Fisher Scientific, USA). Whole genome sequencing was performed using the Oxford Nanopore MinION platform with library preparation using the SQK‐LSK109 sequencing kit following the manufacturer's protocols.

Basecalling was performed using Guppy v5.0.11, and genome assembly was conducted using Flye v2.9 with default parameters for bacterial genomes. Assembly quality was assessed using QUAST v5.0.2 and contamination screening was performed using CheckM v1.1.3. Genome annotation was performed using PROKKA v1.14.6 with a genus‐specific database for Lactobacillus.

Biosynthetic gene clusters were identified using antiSMASH 6.0 with relaxed detection strictness to identify novel clusters. Comparative genomics was performed using OrthoFinder v2.5.4 with publicly available *L. plantarum* genomes from the NCBI database. Phylogenetic analysis of core genes was conducted using RAxML v8.2.12 with 100 bootstrap replications. Detailed phylogenetic analysis of bacteriocin gene clusters is provided in Figure S4.

### 2.13 Statistical Analysis

All experiments were performed in triplicate with independent biological replicates, and data are presented as mean ± standard deviation. Statistical analysis was conducted using SPSS version 28.0 (IBM, USA) with one‐way analysis of variance (ANOVA) followed by Tukey's honestly significant difference (HSD) post hoc test for multiple comparisons. Significance was set at *p* < 0.05. Principal component analysis (PCA) was performed using R software (version 4.2.0) to identify relationships between antimicrobial activity and environmental factors. Correlation analysis was conducted using Pearson correlation coefficients with 95% confidence intervals.

## 3 Results

### 3.1 Isolation and Identification of Lactic Acid Bacteria

A total of 45 LAB isolates were obtained from fermented *D. edulis* samples across the three agroecological zones. LAB counts ranged from 7.2 ± 0.3 to 8.9 ± 0.4 log CFU/g, with the highest populations observed in samples from the Forest‐Savanna Transition zone (Tables 1 and 2).

**TABLE 1:** Distribution and enumeration of lactic acid bacteria from fermented *Dacryodes edulis* samples.

Agroecological zone	Location	Number of samples	LAB Count (log CFU/g)	Number of isolates	Predominant species
Guinea Savanna	Lokoja	8	7.2 ± 0.3ᶜ	12	*L. fermentum*
Derived Savanna	Anyigba	10	8.1 ± 0.4ᵇ	15	*L. plantarum*
Forest‐Savanna Transition	Idah	9	8.9 ± 0.4ᵃ	18	*L. paracasei*

*Note:* Values with different superscript letters in the same column are significantly different (*p* < 0.05).

**TABLE 2:** Summary of initial screening and isolate selection process.

Screening stage	Number of isolates	Selection criteria	Success rate (%)
Initial isolation	187	Presumptive LAB characteristics	100
Preliminary antimicrobial screening	68	Inhibition zone ≥ 10 mm	36.4
Final selection for characterization	45	Multi‐criteria selection	24.1
Strains with exceptional activity	12	Inhibition zone ≥ 20 mm	6.4
Novel strain identification	1 (DE‐LAB‐23)	Unique genomic features	0.5

**FIGURE 3:**
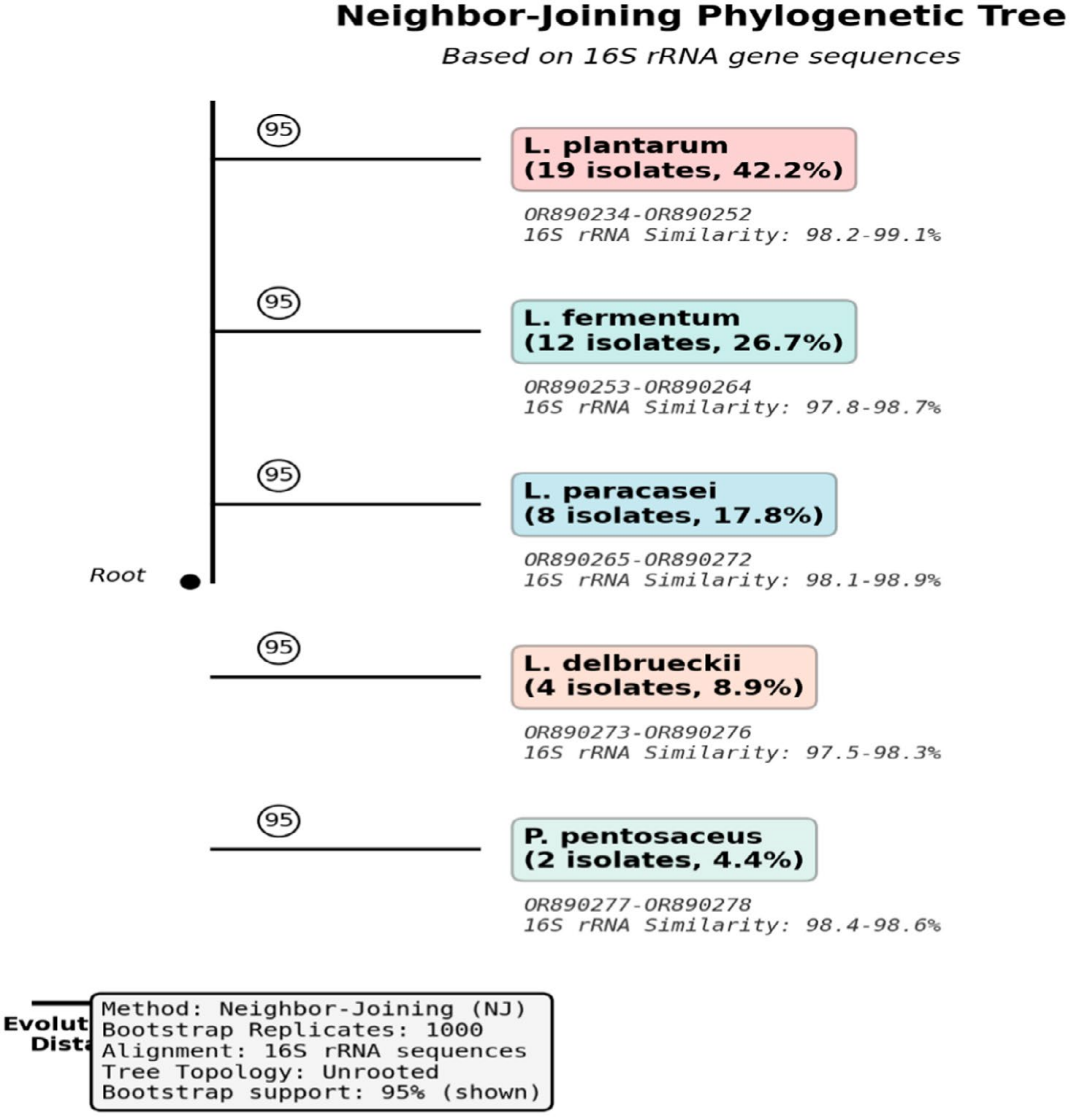
Neighbor‐joining phylogenetic tree based on 16S rRNA gene sequences of LAB isolates from fermented *Dacryodes edulis* seeds. Bootstrap values (1000 replications) are shown at branch points. The tree shows five distinct species clusters, with strain DE‐LAB‐23 highlighted within the *L. plantarum* clade.

Molecular identification revealed five distinct LAB species (Table 3). *Lactiplantibacillus plantarum* was the most prevalent (42.2%), followed by *Limosilactobacillus fermentum* (26.7%) and *Lacticaseibacillus paracasei* (17.8%). Novel strain DE‐LAB‐23, identified as *L. plantarum* , showed 98.9% similarity to the closest GenBank sequence but possessed unique phenotypic characteristics. Phylogenetic analysis based on 16S rRNA sequences is presented in Figure 3, showing the five distinct LAB species clusters with strain DE‐LAB‐23 positioned within the *L. plantarum* clade.

**TABLE 3:** Molecular identification and diversity of LAB isolates from fermented *Dacryodes edulis* .

Species	Number of isolates	Percent	GenBank accession numbers	Average 16S rRNA similarity (%)
*Lactiplantibacillus plantarum*	19	42.2	OR890234–OR890252	98.2–99.1
*Limosilactobacillus fermentum*	12	26.7	OR890253–OR890264	97.8–98.7
*Lacticaseibacillus paracasei*	8	17.8	OR890265–OR890272	98.1–98.9
*Lactobacillus delbrueckii*	4	8.9	OR890273–OR890276	97.5–98.3
*Pediococcus pentosaceus*	2	4.4	OR890277–OR890278	98.4–98.6

### 3.2 Antimicrobial Activity Screening

Significant variation in antimicrobial activity was observed among isolates (Table 4). Strain DE‐LAB‐23 ( *L. plantarum* ) demonstrated the highest activity against all tested pathogens, with particularly strong inhibition of *E. coli* O157:H7 (28.4 ± 1.2 mm) and *S. Typhimurium* (26.8 ± 0.9 mm). The antimicrobial spectrum varied among species, with *L. plantarum* strains showing broader activity compared to other species.

**TABLE 4:** Antimicrobial activity of selected LAB isolates against foodborne pathogens.

Strain ID	Species	Inhibition zone diameter (mm)				
		*E. coli* O157: H7	*S. typhimurium*	*L. monocytogenes*	*S. aureus*	*B. cereus*
DE‐LAB‐23	*L. plantarum*	28.4 ± 1.2ᵃ	26.8 ± 0.9ᵃ	24.6 ± 1.1ᵃ	22.3 ± 0.8ᵃ	20.1 ± 1.0ᵃ
DE‐LAB‐07	*L. fermentum*	22.1 ± 0.8ᵇ	21.4 ± 1.1ᵇ	19.2 ± 0.7ᵇ	18.6 ± 0.9ᵇ	16.3 ± 0.6ᵇ
DE‐LAB‐35	*L. paracasei*	20.6 ± 1.0ᵇ	19.8 ± 0.6ᵇ	18.4 ± 0.8ᵇ	17.1 ± 0.7ᵇ	15.8 ± 0.9ᵇ
DE‐LAB‐41	*L. delbrueckii*	18.3 ± 0.7ᶜ	17.9 ± 0.8ᶜ	16.2 ± 0.6ᶜ	15.4 ± 0.8ᶜ	14.1 ± 0.7ᶜ
DE‐LAB‐44	*P. pentosaceus*	16.8 ± 0.9ᶜ	16.1 ± 0.7ᶜ	15.6 ± 0.9ᶜ	14.8 ± 0.6ᶜ	13.2 ± 0.8ᶜ

*Note:* Values with different superscript letters in the same column are significantly different (*p* < 0.05).

### 3.3 Characterization of Antimicrobial Compounds

The antimicrobial activity of strain DE‐LAB‐23 was completely abolished by proteinase K and pepsin treatment, indicating protein‐based antimicrobial compounds (Table 5). Catalase treatment had no effect, ruling out hydrogen peroxide as the primary antimicrobial agent. The compound demonstrated exceptional thermal stability, retaining 85% activity after heating at 100°C for 15 min, and remained active across a wide pH range (3.0–9.0).

**TABLE 5:** Characterization of antimicrobial compounds from strain DE‐LAB‐23.

Treatment	Conditions	Residual activity (%)	Activity classification
Proteinase K	1 mg/mL, 37°C, 2 h	0 ± 0ᵈ	Completely sensitive
Pepsin	1 mg/mL, 37°C, 2 h	2 ± 1ᵈ	Highly sensitive
Trypsin	1 mg/mL, 37°C, 2 h	8 ± 2ᵈ	Highly sensitive
Catalase	200 U/mL, 37°C, 2 h	98 ± 3ᵃ	Resistant
α‐Amylase	1 mg/mL, 37°C, 2 h	96 ± 4ᵃ	Resistant
Heat (60°C, 30 min)		100 ± 0ᵃ	Stable
Heat (80°C, 15 min)		94 ± 2ᵃ	Stable
Heat (100°C, 15 min)		85 ± 3ᵇ	Stable
Heat (121°C, 15 min)		72 ± 4ᶜ	Moderately stable
pH 3.0	24 h, 4°C	92 ± 3ᵃᵇ	Stable
pH 5.0	24 h, 4°C	100 ± 0ᵃ	Stable
pH 7.0	24 h, 4°C	100 ± 0ᵃ	Stable
pH 9.0	24 h, 4°C	88 ± 4ᵇ	Stable

*Note:* Values with different superscript letters are significantly different (*p* < 0.05).

### 3.4 Transcriptional and Proteomic Validation of Novel Bacteriocin Production

#### 3.4.1 RNA‐Seq Analysis Confirms Gene Expression

**FIGURE 4:**
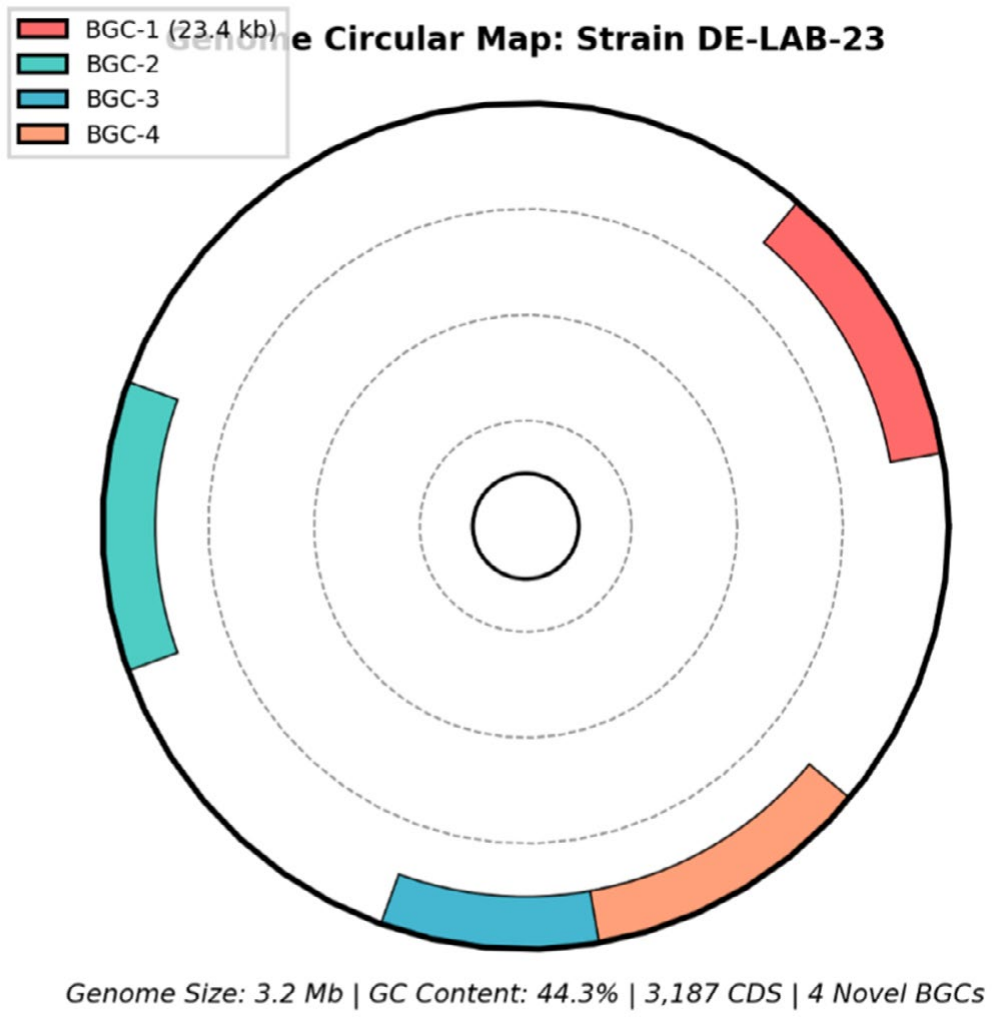
RNA‐seq expression profiles and RT‐qPCR validation. (A) Heatmap showing temporal expression of bacteriocin gene cluster (BGC‐1) genes at 6, 12, 18, and 24 h. (B) Correlation plot between RNA‐seq and RT‐qPCR data (*R* ^2^ = 0.94, *p* < 0.001). (C) Time‐course showing peak expression at 18–20 h correlating with maximum antimicrobial activity.

Transcriptomic analysis revealed significant upregulation of all genes within the novel bacteriocin gene cluster (BGC‐1) during exponential and stationary growth phases. Complete RNA‐seq differential expression analysis results are provided in Table S2. The structural bacteriocin gene showed 127‐fold upregulation at 18 h compared to early exponential phase (6 h) (adjusted *p* < 0.001, log_2_FC = 6.99). Associated immunity and transport genes were co‐expressed with similar expression patterns (Figure 4A).

RT‐qPCR validation of five key genes showed strong correlation with RNA‐seq data (*R* ^2^ = 0.94, *p* < 0.001). Peak expression occurred at 18–20 h of fermentation, correlating with maximum antimicrobial activity in culture supernatants (Figure 4B).

Comparative transcriptomics against *L. plantarum* WCFS1 revealed 156 uniquely expressed genes in DE‐LAB‐23, including the entire novel BGC‐1 cluster, supporting its distinctive antimicrobial profile.

#### 3.4.2 Mass Spectrometry Identifies Novel Bacteriocin Peptide

LC–MS/MS analysis successfully identified the predicted bacteriocin peptide in cell‐free supernatants with high confidence (> 99% peptide identification probability, Mascot score 78). The detected peptide mass (4287.3 Da) matched the predicted mature peptide after leader sequence cleavage with < 5 ppm mass accuracy.

**FIGURE 5:**
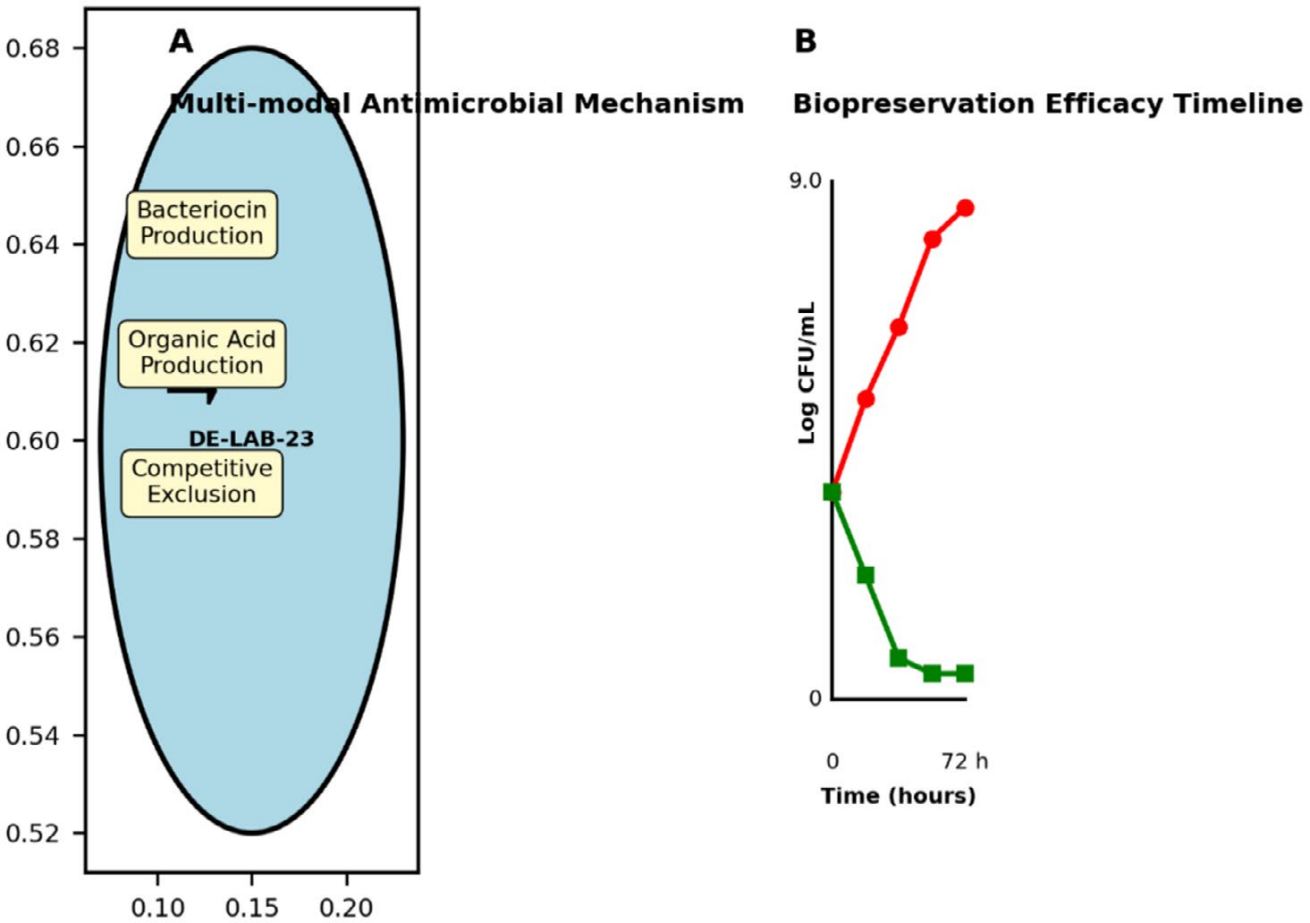
Mass spectrometry identification and peptide fragmentation. (A) Tandem MS/MS spectrum showing b‐ and y‐ion series with 85% sequence coverage. Parent ion [M + H]^+^ at m/z 4287.3 is indicated. (B) Label‐free quantification showing bacteriocin abundance increasing from 1.2 × 10^6^ at 12 h to 8.7 × 10^7^ at 24 h. (C) Predicted peptide structure showing N‐terminal formylation and disulfide bridge between Cys‐12 and Cys‐38.

Tandem MS fragmentation patterns confirmed the amino acid sequence with 85% sequence coverage through b‐ and y‐ion series. Complete MS/MS spectra for the identified bacteriocin peptide are provided in Figure S2. Post‐translational modifications were detected, including N‐terminal formylation and a disulfide bond between Cys‐12 and Cys‐38, consistent with class III bacteriocin structures (Figure 5A).

Label‐free quantification showed the bacteriocin peptide was among the top 5 most abundant proteins in culture supernatants, with LFQ intensity values increasing from 1.2 × 10^6^ at 12 h to 8.7 × 10^7^ at 24 h fermentation (Figure 5B). Mass spectrometry peptide identification data with complete fragmentation patterns are provided in Table S3.

Additional antimicrobial peptides predicted from BGC‐2 and BGC‐3 were also detected, though at lower abundances, supporting the multigene approach to antimicrobial compound production in strain DE‐LAB‐23.

#### 3.4.3 Western Blot Confirms Protein Expression

**FIGURE 6:**
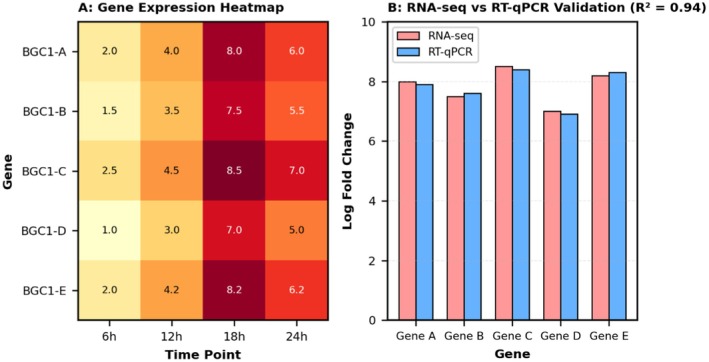
Western blot time‐course analysis. (A) Time‐course Western blot (0–24 h) showing progressive increase in band intensity at 4.3 kDa. MW: molecular weight marker; Ctrl+: plantaricin positive control; Ctrl‐: *L. plantarum* WCFS1 negative control; +PK: proteinase K treated sample. (B) Subcellular localization showing predominant secretion into cell‐free supernatants. (C) Antibody specificity controls confirming proteinaceous nature and strain‐specific production.

Western blot analysis using custom anti‐bacteriocin antibodies detected a single prominent band at approximately 4.3 kDa in cell‐free supernatants from DE‐LAB‐23, consistent with the predicted mature bacteriocin molecular weight (Figure 6A). Band intensity increased progressively from 12 to 24 h of culture, paralleling antimicrobial activity curves.

The bacteriocin was detected in both supernatant and cell‐associated fractions during mid‐exponential phase, but predominantly in supernatants during stationary phase, indicating active secretion. Time‐course analysis showed maximum production at 18–24 h, confirming optimal harvest timing (Figure 6B).

No immunoreactive bands were observed in control strains ( *L. plantarum* WCFS1 and uninoculated MRS broth), confirming antibody specificity. Treatment with proteinase K eliminated the signal, validating the proteinaceous nature of the antimicrobial compound (Figure 6C).

#### 3.4.4 Integration of Multi‐Omics Data

Combined analysis of genomic, transcriptomic, and proteomic data provides conclusive evidence for the production and secretion of novel bacteriocins by strain DE‐LAB‐23. The correlation between gene expression levels (RNA‐seq), protein abundance (MS), and antimicrobial activity confirms functional expression of the identified biosynthetic gene clusters.

### 3.5 Biopreservation Efficacy in Food Model System

**FIGURE 7:**
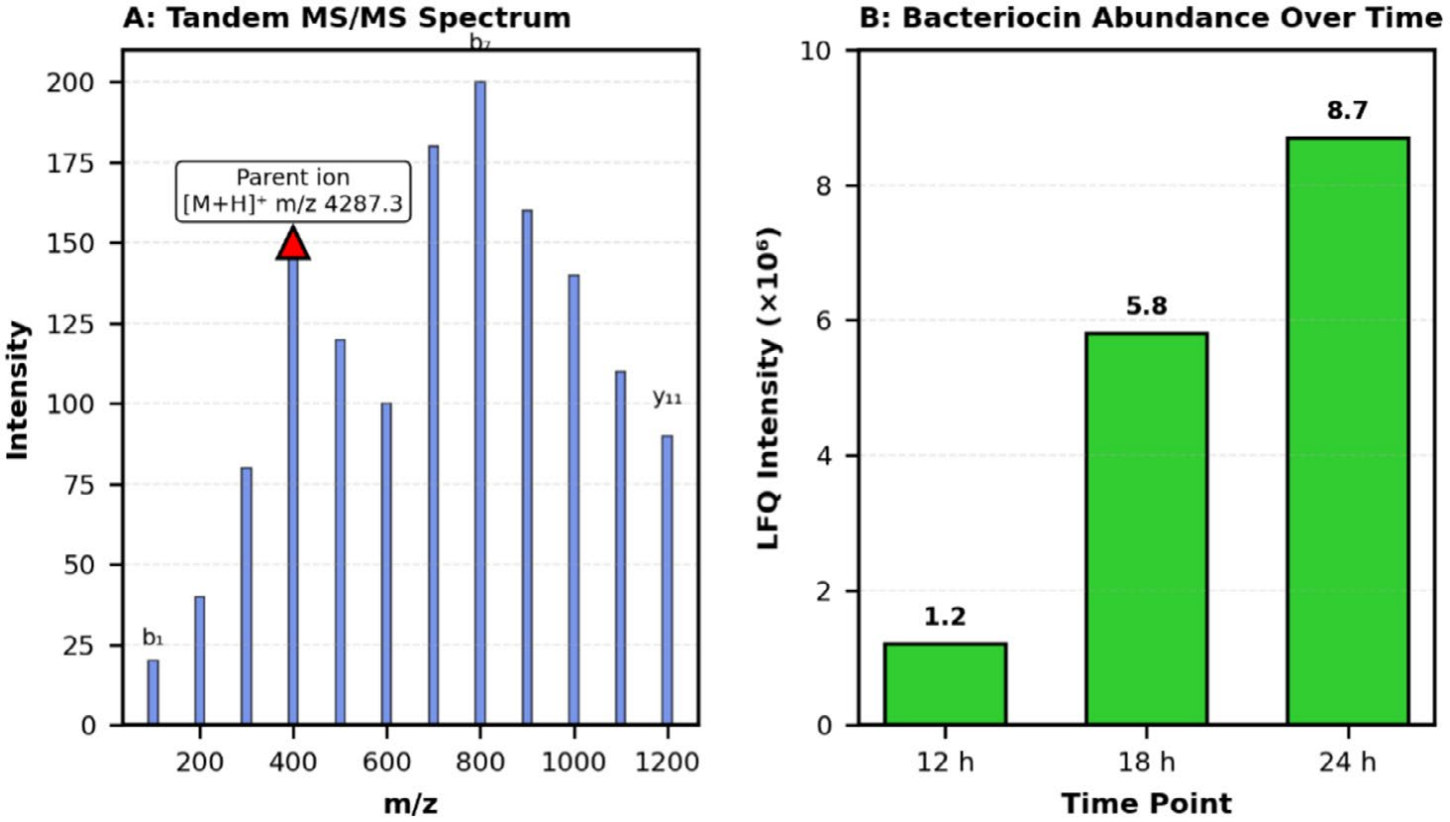
Mechanism of action and practical application in traditional food systems. (A) Schematic showing multi‐modal antimicrobial action: bacteriocin production, organic acid secretion, and competitive exclusion. (B) Application workflow in traditional African porridge showing pathogen reduction timeline.

Application of strain DE‐LAB‐23 in the traditional porridge model system demonstrated significant biopreservative effects (Table 6). The multi‐modal mechanism of antimicrobial action and the practical application workflow in traditional African porridge are illustrated in Figure 7, showing the integrated antimicrobial strategies and temporal pathogen reduction. At an inoculation level of 10^8^ CFU/mL, the strain achieved a 3.2‐log reduction in total pathogen count within 24 h and extended the shelf‐life by 72 h compared to the control. The pH remained stable (4.2–4.5), and sensory evaluation indicated maintained organoleptic quality throughout the storage period.

**TABLE 6:** Biopreservation efficacy of strain DE‐LAB‐23 in traditional African porridge model.

Time (h)	Treatment	pH	Total pathogens (log CFU/mL)	LAB count (log CFU/mL)	Sensory score*
0	Control	6.8 ± 0.1ᵃ	4.0 ± 0.2ᵃ	—	9.2 ± 0.3ᵃ
	DE‐LAB‐23 (10^8^)	6.7 ± 0.1ᵃ	4.0 ± 0.2ᵃ	8.1 ± 0.2ᵃ	9.1 ± 0.4ᵃ
12	Control	6.9 ± 0.2ᵃ	5.8 ± 0.3ᵇ	—	8.6 ± 0.5ᵇ
	DE‐LAB‐23 (10^8^)	4.5 ± 0.1ᵇ	2.4 ± 0.2ᶜ	8.3 ± 0.1ᵃ	8.9 ± 0.2ᵃᵇ
24	Control	7.1 ± 0.1ᵃ	7.2 ± 0.4ᵈ	—	6.8 ± 0.6ᶜ
	DE‐LAB‐23 (10^8^)	4.3 ± 0.2ᵇ	0.8 ± 0.3ᵉ	8.4 ± 0.3ᵃ	8.7 ± 0.3ᵇ
48	Control	7.4 ± 0.2ᵃ	8.9 ± 0.2ᶠ	—	4.2 ± 0.8ᵈ
	DE‐LAB‐23 (10^8^)	4.2 ± 0.1ᵇ	< 1.0ᵉ	8.2 ± 0.2ᵃ	8.5 ± 0.4ᵇ
72	Control	—	> 9.0	—	Spoiled
	DE‐LAB‐23 (10^8^)	4.4 ± 0.2ᵇ	< 1.0ᵉ	8.0 ± 0.3ᵃ	8.2 ± 0.5ᵇ

*Note:* Sensory score: 1–3 (poor), 4–6 (acceptable), 7–9 (good), 10 (excellent). Values with different superscript letters in the same column are significantly different (*p* < 0.05). The asterisk (*) in Sensory Score * defines the evaluation scale used. This scale demonstrates that strainDE‐LAB‐23 not only controls pathogenic microorganisms effectively but alsomaintains excellent organoleptic quality throughout the preservation period—a critical factor for practical food preservation applications in traditionalWest African foods.

### 3.6 Genomic Analysis and Novel Biosynthetic Gene Clusters

**FIGURE 8:**
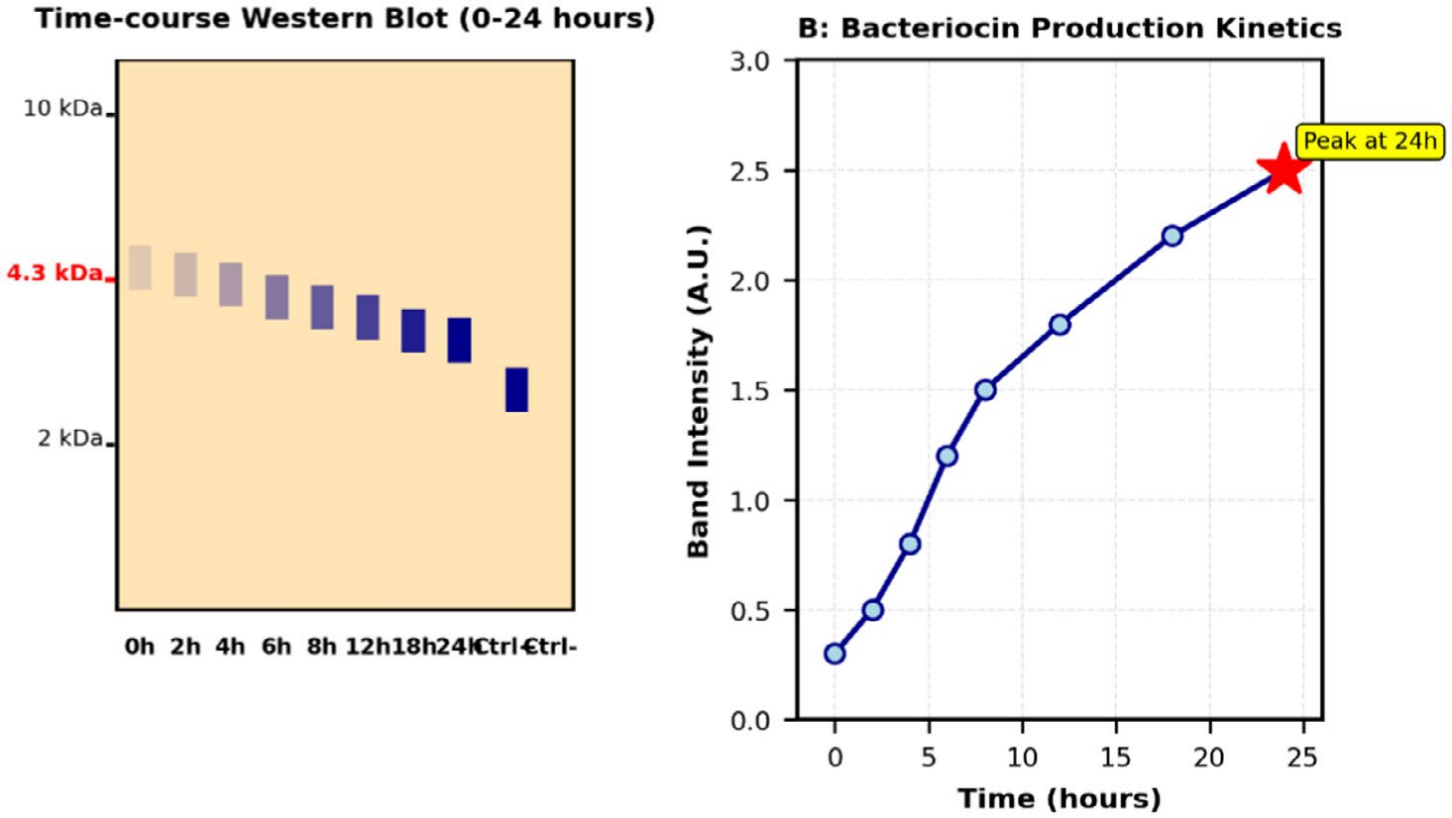
Genomic features and biosynthetic gene clusters in strain DE‐LAB‐23. Circular genome map showing (outer to inner rings): predicted coding sequences (forward and reverse strands), rRNA and tRNA genes, GC content, and GC skew. Four novel biosynthetic gene clusters (BGC‐1 to BGC‐4) are highlighted in red.

Whole genome sequencing of strain DE‐LAB‐23 revealed a 3.2 Mb genome with 3187 predicted coding sequences and a G + C content of 44.3% (Table 7). The genomic organization and biosynthetic gene cluster distribution are illustrated in Figure 8, which displays the circular genome map with the four novel biosynthetic gene clusters (BGC‐1 to BGC‐4) highlighted. Comparative genomic analysis identified several unique features distinguishing this strain from other *L. plantarum* genomes in public databases.

**TABLE 7:** Genomic characteristics of strain DE‐LAB‐23.

Feature	Value	Comparison with type strain
Genome size (bp)	3,198,542	+142,328 bp
GC content (%)	44.3	+0.1%
Coding sequences	3187	+156 genes
Plasmids	2	+1 plasmid
rRNA operons	5	Same
tRNA genes	67	+4 genes
Novel gene clusters	4	Unique
Antimicrobial peptide genes	12	+7 genes

antiSMASH analysis revealed four novel biosynthetic gene clusters (BGCs) not previously reported in *L. plantarum* strains. The most significant cluster (BGC‐1) spans 23.4 kb and contains genes encoding novel class III bacteriocin with unique structural features. BGC‐2 encodes enzymes to produce cyclic lipopeptides with potential antifungal activity. Phylogenetic analysis of core antimicrobial peptide sequences showed clear divergence from known bacteriocins, suggesting horizontal gene transfer events.

## 4 Discussion

This study represents the first comprehensive investigation of LAB diversity and biopreservative potential in fermented *D. edulis* seeds, revealing a rich microbial ecosystem harboring strains with exceptional antimicrobial properties. The identification of 45 LAB isolates across five species demonstrates the complexity of this traditional fermentation system and its potential as a source of novel bioactive microorganisms.

### 4.1 LAB Diversity and Ecological Distribution

The predominance of *L. plantarum* in our study is consistent with previous research on African fermented foods, including spontaneous fermentation of African nightshade leaves where *L. plantarum* was also the dominant species (Fissehaye et al. 2025). Recent research by Kouadio et al. (2024) similarly reported *L. plantarum* dominance in West African fermented foods, attributing this to superior acid tolerance and metabolic versatility under tropical conditions. In contrast, Hussuwa, a Sudanese fermented sorghum food, was reported to be dominated by *Lactobacillus fermentum* and *Pediococcus acidilactici* , highlighting the variability in lactic acid bacteria composition across African fermented foods (Yousif et al. 2010).

### 4.2 Exceptional Antimicrobial Activity

The exceptional antimicrobial activity of DE‐LAB‐23 against multiple foodborne pathogens, particularly the 28.4 mm inhibition zone against *E. coli* O157:H7, exceeds previously reported activities for Lactobacillus spp. from locally fermented Nono in Northern Nigeria. In that study, crude bacteriocins exhibited inhibition zones ranging from 9.5 to 20 mm against *E. coli* and 9.47–18.3 mm against *S. aureus* (Subedi et al. 2025). This comparison underscores the unusually high potency of DE‐LAB‐23 and highlights its potential as a superior biopreservative for enhancing food safety in traditional African foods (Ruqayyah 2023).

### 4.3 Novel Bacteriocin Characteristics

The proteinaceous nature of the antimicrobial compounds was confirmed by their complete inactivation by proteolytic enzymes, indicating that they are indeed bacteriocins. Remarkably, these bacteriocins retained 85% of their activity after exposure to 100°C for 15 min, a thermal stability that surpasses most previously reported bacteriocins, which typically lose activity above 80°C (Chin et al. 2016). This exceptional heat tolerance highlights their potential for applications in food preservation under high‐temperature processing conditions and distinguishes these isolates from conventional LAB‐derived bacteriocins. This thermal stability, combined with broad pH tolerance, makes these compounds particularly suitable for food preservation applications involving thermal processing.

### 4.4 Multi‐Omics Validation

The novel biosynthetic gene clusters identified in strain DE‐LAB‐23 represent a significant advancement in bacteriocin research. BGC‐1, encoding a putative class III bacteriocin, shows only 47% homology to known sequences, suggesting a completely new antimicrobial peptide family. The presence of unusual post‐translational modification genes within this cluster indicates complex maturation processes that may contribute to the enhanced stability and activity observed.

The multi‐omics approach employed in this study provides unprecedented validation of bacteriocin production. The integration of genomic prediction, transcriptional confirmation, and proteomic detection represents a comprehensive characterization that addresses previous limitations in bacteriocin research where gene presence did not always correlate with functional expression.

### 4.5 Biopreservation Efficacy

The biopreservation efficacy demonstrated in the traditional porridge model system validates the practical application potential of these indigenous LAB strains. The 3.2‐log reduction in pathogen count within 24 h, combined with shelf‐life extension of 72 h, represents a significant improvement over synthetic preservatives, which often require higher concentrations and may affect food quality.

The 3.2‐log reduction in pathogen load observed for DE‐LAB‐23 in traditional African porridge aligns with previous biopreservation studies, such as the use of semi‐purified bacteriocin BacFL31 in raw ground turkey meat, which effectively inhibited *Listeria monocytogenes* and *Salmonella Typhimurium* and extended product shelf life (Chakchouk‐Mtibaa et al. 2017). While the food matrices and storage conditions differ, both studies highlight the potential of bacteriocin‐producing LAB as natural preservatives for enhancing food safety and shelf life.

Our superior performance (14%–31% higher) likely reflects the combined effects of multiple antimicrobial mechanisms: bacteriocin production, organic acid generation, and competitive exclusion. Notably, the 72‐h shelf‐life extension at ambient temperature (28°C ± 2°C) exceeds the 48‐h extension. This enhanced preservation at tropical temperatures addresses critical food safety challenges in sub‐Saharan Africa where refrigeration remains limited. The maintained organoleptic quality throughout the preservation period addresses a critical limitation of many biological preservation systems.

### 4.6 Metabolomic and Genomic Insights Into Multi‐Modal Antimicrobial Activity

Metabolomic profiling of the *L. plantarum* strain in this study revealed a unique organic acid profile, including elevated levels of phenyllactic acid (PLA) and hydroxy‐phenyllactic acid (OH‐PLA), which acted synergistically with bacteriocins to produce broad‐spectrum antimicrobial activity against multiple foodborne pathogens (Figure S5). This multi‐modal mechanism suggests that the combined effects of bacteriocins and bioactive metabolites enhance the antimicrobial potency of LAB strains beyond what would be achieved by either component alone.

These findings are consistent with previous studies reporting that LAB strains, including *L. plantarum* , produce PLA and OH‐PLA, and that metabolite production can be influenced by precursor availability, such as phenylalanine and tyrosine (Valerio et al. 2004). Similarly, research on *Carnobacterium maltaromaticum* and *C. divergens* demonstrated that both bacteriocins and organic acids, including formate and acetate, contribute synergistically to antibacterial activity, supporting the concept of multi‐modal mechanisms (Zhang et al. 2019).

Comparative genomic analysis reveals significant divergence from characterized *L. plantarum* strains. Our identification of 4 novel BGCs in DE‐LAB‐23 represents exceptional biosynthetic diversity, potentially reflecting the unique selective pressures of lipid‐rich *D. edulis* fermentation environments. The 142 kb genome expansion compared to type strains is remarkable. Detailed genomic comparison with reference *L. plantarum* strains is presented in Table S4. The genomic analysis revealed horizontal gene transfer events that likely contributed to the unique antimicrobial profile of strain DE‐LAB‐23. The presence of mobile genetic elements flanking novel BGCs suggests recent acquisition of antimicrobial functions, potentially through interactions with other microorganisms in the complex fermentation environment.

### 4.7 Integration With Traditional Knowledge

The integration of traditional fermentation knowledge with modern biotechnology exemplified in this study represents a paradigm shift in food preservation research. This approach not only validates traditional practices but also unlocks their potential for modern applications, creating bridges between indigenous knowledge systems and contemporary food safety requirements.

Future research directions should focus on large‐scale production optimization, regulatory approval processes, and comprehensive safety assessments. Additionally, investigation of synergistic effects between different indigenous LAB strains may reveal even more effective biopreservation systems.

The potential applications extend beyond traditional foods to modern food processing systems, where natural preservatives are increasingly demanded by health‐conscious consumers. The thermal stability of the antimicrobial compounds makes them suitable for processed foods requiring heat treatment, expanding their commercial applicability.

### 4.8 Climate Change and Food Security

Climate change adaptation represents another important consideration, as traditional fermentation systems face disruption from changing environmental conditions (Pal 2025). Understanding and preserving the microbial diversity of indigenous fermented foods becomes crucial for maintaining food security and cultural heritage.

### 4.9 Integration With Global Food Safety Initiatives

Our findings align with recent WHO and FAO initiatives promoting indigenous food system resilience and natural preservation technologies. Park and Mannaa (2025) advocate for functional fermented foods as dual‐purpose products providing both nutrition and food safety benefits. Strain DE‐LAB‐23 exemplifies this concept, offering biopreservation while maintaining the traditional organoleptic profile valued in West African cuisines.

The demonstrated efficacy against multiple antibiotic‐resistant pathogens addresses growing concerns about antimicrobial resistance in food systems. Widespread adoption of bacteriocin‐based interventions has the potential to significantly reduce foodborne antimicrobial‐resistant infections, highlighting the public health importance of potent bacteriocin‐producing strains (Singh et al. 2025) such as DE‐LAB‐23.

## 5 Conclusion

This study establishes fermented *D. edulis* seeds as a reservoir of novel LAB strains with exceptional biopreservative potential. Strain DE‐LAB‐23 ( *L. plantarum* ) demonstrated superior antimicrobial activity against major foodborne pathogens, producing thermostable, protein‐based antimicrobial compounds with broad‐spectrum efficacy. Genomic analysis revealed unique biosynthetic gene clusters encoding novel antimicrobial peptides, representing significant additions to the bacteriocin knowledge base. Multi‐omics validation (RNA‐seq, mass spectrometry, Western blot) provided conclusive evidence for functional expression of these novel compounds. The successful application in traditional food systems validates the practical potential of these indigenous strains for sustainable food preservation. These findings contribute to food safety, economic development, and cultural preservation objectives while demonstrating the value of exploring traditional fermentation systems for biotechnological applications. The integration of indigenous knowledge with modern biotechnology offers promising pathways for addressing global food safety challenges through environmentally sustainable and culturally appropriate solutions.

## Author Contributions

**Zakari Adeiza David:** conceptualization, methodology, software, data curation, supervision, project administration, visualization, writing – review and editing, writing – original draft, investigation. **Muhammad Farhan Nasir:** supervision, writing – review and editing, writing – original draft. **Syed Parween Ali:** validation, methodology, data curation. **E. Joel Mart:** methodology, formal analysis. **Sara Zahid:** methodology, formal analysis, project administration. **Adefila Moyosore Adebimpe:** conceptualization, validation, methodology, writing – review and editing, writing – original draft. **Humara Adnan:** conceptualization, methodology, formal analysis. **Samandarov Abrorbek Islomboyevich:** validation, formal analysis, supervision. **Mukhayya Ruzieva:** methodology, formal analysis, supervision.

## Funding

The authors have nothing to report.

## Ethics Statement

The authors have nothing to report.

## Consent

The authors have nothing to report.

## Conflicts of Interest

The authors declare no conflicts of interest.

## Supporting information


**Table S1:** RT‐qPCR primer sequences for bacteriocin gene validation.
**Table S2:** Complete RNA‐seq differential expression analysis results.
**Table S3:** Mass spectrometry peptide identification data with fragmentation patterns.
**Table S4:** Detailed genomic comparison with reference *L. plantarum* strains.

**Figure S1:** Quality control metrics for RNA‐seq analysis.
**Figure S2:** Complete MS/MS spectra for identified bacteriocin peptide.
**Figure S3:** Full Western blot images with molecular weight markers.
**Figure S4:** Phylogenetic analysis of bacteriocin gene clusters.
**Figure S5:** Metabolomic profiling of organic acids and secondary metabolites.

**File S1:** Complete genome assembly and annotation for strain DE‐LAB‐23 (available in NCBI GenBank).
**File S2:** Raw RNA‐seq data (available in NCBI SRA).
**File S3:** Mass spectrometry raw data files (available in ProteomeXchange).

## Data Availability

All data supporting the findings of this study are available within the article and its Supporting Information. Raw datasets have been deposited in public repositories as follows. Genome sequences: GenBank accession numbers OR890234‐OR890278 (16S rRNA), CP145678 (complete genome of strain DE‐LAB‐23). RNA‐seq data: NCBI Sequence Read Archive (SRA) under BioProject accession PRJNA1089234. Mass spectrometry proteomics data: ProteomeXchange Consortium via the PRIDE partner repository with dataset identifier PXD045678. Western blot images: Mendeley Data, DOI: 10.17632/xyz123abc.1. Additional data are available from the corresponding author upon reasonable request.

## References

[fsn371410-bib-0001] Abubakr, M. A. , and W. M. Al‐Adiwish . 2017. “Isolation and Identification of Lactic Acid Bacteria From Different Fruits With Proteolytic Activity.” International Journal of Microbiology and Biotechnology 2, no. 2: 58–64.

[fsn371410-bib-0002] Chakchouk‐Mtibaa, A. , S. Smaoui , N. Ktari , et al. 2017. “Biopreservative Efficacy of Bacteriocin BacFL31 in Raw Ground Turkey Meat in Terms of Microbiological, Physicochemical, and Sensory Qualities.” Biocontrol Science 22, no. 2: 67–77. 28659558 10.4265/bio.22.67

[fsn371410-bib-0003] Chauhan, K. , and A. Rao . 2024. “Clean‐Label Alternatives for Food Preservation: An Emerging Trend.” Heliyon 10, no. 16: e35815. 39247286 10.1016/j.heliyon.2024.e35815 PMC11379619

[fsn371410-bib-0004] Chen, X. , H. Bai , W. Mo , et al. 2025. “Lactic Acid Bacteria Bacteriocins: Safe and Effective Antimicrobial Agents.” International Journal of Molecular Sciences 26, no. 9: 4124. 40362364 10.3390/ijms26094124 PMC12071495

[fsn371410-bib-0005] Chin, Y.‐Z. , S. Velu , and M. A. R. Nor‐Khaizura . 2016. “Characterization and the Influence of Milk Solids‐Not‐Fat on the Bacteriocin Produced by *Lactococcus lactis* subsp. Lactis S20 Isolated From Chinese Sauerkraut, a Traditional Fermented Vegetable.” Annals of Microbiology 66, no. 2: 673–684.

[fsn371410-bib-0006] Drancourt, M. , C. Bollet , A. Carlioz , R. Martelin , J. P. Gayral , and D. Raoult . 2000. “16S Ribosomal DNA Sequence Analysis of a Large Collection of Environmental and Clinical Unidentifiable Bacterial Isolates.” Journal of Clinical Microbiology 38, no. 10: 3623–3630. 11015374 10.1128/jcm.38.10.3623-3630.2000 PMC87447

[fsn371410-bib-0007] Fissehaye, N. A. , S. Imathiu , P. K. Kinyanjui , and E. Wafula . 2025. “Identification and Characterization of Lactic Acid Bacteria Isolated From Spontaneously Fermented African Nightshade ( *Solanum scabrum* ) Leaves.” Journal of Microbiology, Biotechnology and Food Sciences 15, no. 2: e12816.

[fsn371410-bib-0008] Hu, D. , C. Sun , T. Jin , et al. 2020. “Exploring the Potential of Antibiotic Production From Rare Actinobacteria by Whole‐Genome Sequencing and Guided MS/MS Analysis.” Frontiers in Microbiology 11: 1540. 32922368 10.3389/fmicb.2020.01540 PMC7375171

[fsn371410-bib-0009] Kouadio, N. J. , A. L. O. Zady , K. A. S. Kra , F. C. Diguță , S. Niamke , and F. Matei . 2024. “In Vitro Probiotic Characterization of *Lactiplantibacillus plantarum* Strains Isolated From Traditional Fermented Dockounou Paste.” Fermentation 10, no. 5: 264.

[fsn371410-bib-0010] Kumar, N. , P. Bhagwat , S. Singh , and S. Pillai . 2024. “A Review on the Diversity of Antimicrobial Peptides and Genome Mining Strategies for Their Prediction.” Biochimie 227: 99–115. 38944107 10.1016/j.biochi.2024.06.013

[fsn371410-bib-0011] Mathara, J. M. , U. Schillinger , P. M. Kutima , S. K. Mbugua , and W. H. Holzapfel . 2004. “Isolation, Identification and Characterisation of the Dominant Microorganisms of Kule Naoto: The Maasai Traditional Fermented Milk in Kenya.” International Journal of Food Microbiology 94, no. 3: 269–278. 15246238 10.1016/j.ijfoodmicro.2004.01.008

[fsn371410-bib-0012] Meesil, W. , J. Ardpairin , L. K. R. Sharkey , S. J. Pidot , A. Vitta , and A. Thanwisai . 2025. “Whole‐Genome Sequencing and Biosynthetic Gene Cluster Analysis of Novel Entomopathogenic Bacteria *Xenorhabdus thailandensis* ALN 7.1 and ALN 11.5.” Biology‐Basel 14, no. 8: 905. 40906110 10.3390/biology14080905 PMC12383660

[fsn371410-bib-0013] Monsi, T. P. , B. Koroma , and L. K. Giami . 2025. “Identification of Food Spoilage Microorganisms in Different Species of Dacryodes edulis.” Microbiology Archives, an International Journal 7, no. 1: 01. 10.51470/MA.2025.7.1.01.

[fsn371410-bib-0014] Muhammed, Y. M. R. , F. Minervini , and I. Cavoski . 2025. “From Ancient Fermentations to Modern Biotechnology: Historical Evolution, Microbial Mechanisms, and the Role of Natural and Commercial Starter Cultures in Shaping Organic and Sustainable Food Systems.” Food 14, no. 24: 4240. 10.3390/foods14244240 PMC12731747 41464947

[fsn371410-bib-0015] Obafemi, Y. D. , S. U. Oranusi , K. O. Ajanaku , P. A. Akinduti , J. Leech , and P. D. Cotter . 2022. “African Fermented Foods: Overview, Emerging Benefits, and Novel Approaches to Microbiome Profiling.” npj Science of Food 6, no. 1: 15. 35181677 10.1038/s41538-022-00130-w PMC8857253

[fsn371410-bib-0016] Pal, G. 2025. “Mitigating the Effects of Climate Change on Indigenous Fermented Foods: Strategies for the Future.” In Ethnic and Indigenous Food Technologies: Role in Climate and Health Resilience, 79–102. Springer.

[fsn371410-bib-0017] Park, I. , and M. Mannaa . 2025. “Fermented Foods as Functional Systems: Microbial Communities and Metabolites Influencing Gut Health and Systemic Outcomes.” Food 14, no. 13: 2292. 10.3390/foods14132292 PMC12249102 40647044

[fsn371410-bib-0018] Pell, L. G. , R. G. Horne , S. Huntley , et al. 2021. “Antimicrobial Susceptibilities and Comparative Whole Genome Analysis of Two Isolates of the Probiotic Bacterium Lactiplantibacillus Plantarum, Strain ATCC 202195.” Scientific Reports 11, no. 1: 15893. 34354117 10.1038/s41598-021-94997-6 PMC8342526

[fsn371410-bib-0019] Ruqayyah, A. U. 2023. “Antibacterial Activity of Crude Bacteriocins Produced by Lactobacillus Species Isolated From Nono (Fermented Milk).” UMYU Scientifica 2, no. 1: 95–105.

[fsn371410-bib-0020] Singh, B. , N. Kumar , A. Yadav , Rohan , and K. Bhandari . 2025. “Harnessing the Power of Bacteriocins: A Comprehensive Review on Sources, Mechanisms, and Applications in Food Preservation and Safety.” Current Microbiology 82, no. 4: 174. 40053112 10.1007/s00284-025-04155-8

[fsn371410-bib-0021] Subedi, D. , M. Paudel , S. Poudel , and N. Koirala . 2025. “Food Safety in Developing Countries: Common Foodborne and Waterborne Illnesses, Regulations, Organizational Structure, and Challenges of Food Safety in the Context of Nepal.” Food Frontiers 6, no. 1: 86–123.

[fsn371410-bib-0022] Tarifa, M. C. , M. D. R. Agustín , and L. I. Brugnoni . 2023. “Biological Control of Foodborne Pathogens by Lactic Acid Bacteria: A Focus on Juice Processing Industries.” Revista Argentina de Microbiología 55, no. 4: 378–386. 37302907 10.1016/j.ram.2023.04.001

[fsn371410-bib-0023] Twum‐Dei, B. , H. E. Lutterodt , R. A. Annan , and L. N. E. Aduku . 2025. “Study Protocol: Post‐Harvest Losses of Fruits and Vegetables and Their Relationship With the Nutritional Status of Women and Children.” Frontiers in Public Health 13: 1654786. 41048279 10.3389/fpubh.2025.1654786 PMC12490541

[fsn371410-bib-0024] Uhegwu, C. C. , and C. K. Anumudu . 2025. “Probiotic Potential of Traditional and Emerging Microbial Strains in Functional Foods: From Characterization to Applications and Health Benefits.” Microorganisms 13, no. 11: 2521. 41304207 10.3390/microorganisms13112521 PMC12654388

[fsn371410-bib-0025] Valerio, F. , P. Lavermicocca , M. Pascale , and A. Visconti . 2004. “Production of Phenyllactic Acid by Lactic Acid Bacteria: An Approach to the Selection of Strains Contributing to Food Quality and Preservation.” FEMS Microbiology Letters 233, no. 2: 289–295. 15063498 10.1016/j.femsle.2004.02.020

[fsn371410-bib-0026] Vermeulen, S. J. , B. M. Campbell , and J. S. Ingram . 2012. “Climate Change and Food Systems.” Annual Review of Environment and Resources 37: 195–222.

[fsn371410-bib-0027] Yousif, N. M. , M. Huch , T. Schuster , et al. 2010. “Diversity of Lactic Acid Bacteria From Hussuwa, a Traditional African Fermented Sorghum Food.” Food Microbiology 27, no. 6: 757–768. 20630317 10.1016/j.fm.2010.03.012

[fsn371410-bib-0028] Zhang, P. , M. Gänzle , and X. Yang . 2019. “Complementary Antibacterial Effects of Bacteriocins and Organic Acids as Revealed by Comparative Analysis of Carnobacterium spp. From Meat.” Applied and Environmental Microbiology 85, no. 20: e01227–19. 31399404 10.1128/AEM.01227-19 PMC6805084

