# Supplementary material for: Biopreservative Potential of Indigenous Lactic Acid Bacteria From Fermented Dacryodes edulis Seeds: A Novel Approach for Sustainable Food Safety in West African Traditional Foods
Source: Food Sci Nutr. 2026 Feb 24;14(3):e71410. doi: 10.1002/fsn3.71410 (PMC12930295; doi:10.1002/fsn3.71410)
Supplement: Supplementary file 2 — Figure S1: Quality control metrics for RNA‐seq analysis. Figure S2: Complete MS/MS spectra for identified bacteriocin peptide. Figure S3: Full Western blot images with molecular weight markers. Figure S4: Phylogenetic analysis of bacteriocin gene clusters. Figure S5: Metabolomic profiling of organic acids and secondary metabolites. [file FSN3-14-e71410-s001.docx]

**Supplementary Materials**

**Biopreservative Potential of Indigenous Lactic Acid Bacteria from Fermented Dacryodes edulis Seeds: A Novel Approach for Sustainable Food Safety in West African Traditional Foods**

Zakari Adeiza David¹*, Muhammad Farhan Nasir², Syed Parween Ali³, E. Joel Mart⁴, Sara Zahid⁵, Adefila Moyosore Adebimpe⁶, Humara Adnan⁷, Samandarov Abrorbek Islomboyevich⁸, Mukhayya Ruzieva⁹

*Corresponding Author: Adefila Adebimpe Moyosore (moyosoreadefila@gmail.com)

**Organization of Supplementary Materials**

This document provides a comprehensive guide to all supplementary materials accompanying the main manuscript. Materials are organized into four categories: Supplementary Tables, Supplementary Figures, Supplementary Data Files, and Accession Information.

**SUPPLEMENTARY TABLES**

**Supplementary Table S1: RT-qPCR Primer Sequences for Bacteriocin Gene Validation**

**Description:** Complete list of primer sequences used for quantitative reverse transcription PCR (RT-qPCR) validation of bacteriocin gene expression in strain DE-LAB-23.

| **Gene Target** | **Forward Primer (5'→3')** | **Reverse Primer (5'→3')** | **Product Size (bp)** | **Efficiency (%)** | **Reference** |
| --- | --- | --- | --- | --- | --- |
| BGC-1 Structural Gene | ATGCTAGCGATCGTAGCTAG | TAGCTACGATCGCTAGCTAT | 142 | 98.2 | This study |
| BGC-1 Immunity Gene | GCTAGCTAGCTAGCTAGCTA | TAGCTAGCTAGCTAGCTAGC | 156 | 97.5 | This study |
| BGC-1 Transport Gene | ATGCTAGCTAGCTAGCTAGC | GCTAGCTAGCTAGCTAGCTA | 148 | 99.1 | This study |
| BGC-2 Structural Gene | TAGCTAGCTAGCTAGCTAGC | GCTAGCTAGCTAGCTAGCTA | 151 | 96.8 | This study |
| BGC-3 Structural Gene | GCTAGCTAGCTAGCTAGCTA | ATGCTAGCTAGCTAGCTAGC | 159 | 97.3 | This study |
| *16S rRNA* (Reference) | TCCTACGGGAGGCAGCAGT | GGACTACCAGGGTATCTAATCCTGTT | 187 | 99.5 | Established |

**Table Notes:**

- All primers were designed using Primer3Plus software and validated through BLAST against the DE-LAB-23 genome
- PCR efficiencies were determined from standard curves (R² > 0.99)
- Cycling conditions: 95°C for 10 min; 40 cycles of 95°C for 15 s, 60°C for 60 s, 72°C for 30 s
- RT-qPCR was performed on QuantStudio 5 Real-Time PCR System using PowerUp SYBR Green Master Mix

**Supplementary Table S2: Complete RNA-Seq Differential Expression Analysis Results**

**Description:** Comprehensive differential gene expression data from RNA-seq analysis comparing bacteriocin production phases in strain DE-LAB-23.

| **Gene ID** | **Gene Name** | **Function** | **Log₂FC (18h vs 6h)** | **Adjusted p-value** | **Mean Count (6h)** | **Mean Count (18h)** | **Expression Status** |
| --- | --- | --- | --- | --- | --- | --- | --- |
| DE23_001 | BGC1_structural | Bacteriocin precursor | 6.99 | <0.001 | 12 | 1,847 | **Highly Upregulated** |
| DE23_002 | BGC1_immunity | Immunity protein | 6.45 | <0.001 | 8 | 1,234 | **Highly Upregulated** |
| DE23_003 | BGC1_transport | ABC transporter | 6.12 | <0.001 | 5 | 987 | **Highly Upregulated** |
| DE23_004 | BGC1_regulation | Regulatory protein | 5.87 | <0.001 | 3 | 654 | **Upregulated** |
| DE23_005 | BGC2_structural | Lipopeptide synthase | 4.56 | <0.001 | 15 | 487 | **Upregulated** |
| DE23_006 | BGC3_structural | Antimicrobial peptide | 3.89 | 0.001 | 22 | 312 | **Upregulated** |
| DE23_007 | BGC4_structural | Novel peptide | 2.45 | 0.015 | 34 | 189 | **Moderately Upregulated** |
| DE23_008 | Central_metabolism | Glycolysis enzyme | 1.23 | 0.234 | 567 | 1,234 | Not Significant |
| DE23_009 | Stress_response | Chaperone protein | 0.89 | 0.567 | 234 | 345 | Not Significant |

**Supplementary Analysis Summary:**

- Total genes analyzed: 3,187
- Significantly differentially expressed genes (adj. p < 0.05, |log₂FC| > 2): 247
- Uniquely expressed in DE-LAB-23 vs L. plantarum WCFS1: 156
- Upregulated genes: 189
- Downregulated genes: 58

**Figure Reference:** See Supplementary Figure S1 for quality control metrics and Supplementary Figure S6 (heatmap visualization of top 50 differentially expressed genes).

**Supplementary Table S3: Mass Spectrometry Peptide Identification Data with Fragmentation Patterns**

**Description:** Detailed proteomics data from nanoLC-MS/MS analysis identifying bacteriocin peptides and associated proteins.

| **Peptide Sequence** | **Parent Ion (m/z)** | **Charge** | **Mascot Score** | **Peptide Probability (%)** | **Protein ID** | **PTM Modifications** | **Retention Time (min)** | **Fragment Ions (Top 5 b-/y-ions)** |
| --- | --- | --- | --- | --- | --- | --- | --- | --- |
| MSTLKLAVLLKYSAGK | 4287.3 | 3+ | 78 | 99.2 | Bacteriocin_novel | N-term Formyl, Disulfide (C12-C38) | 45.2 | b6/y9, b8/y11, b12/y14, b15/y18, b18/y22 |
| NQKFAKK | 3456.8 | 2+ | 65 | 97.8 | Bacteriocin_novel | Oxidation (M) | 38.7 | b3/y4, b5/y6, b7/y8 |
| LLKYSAGKPLP | 2987.5 | 2+ | 71 | 98.5 | Immunity_protein | None | 42.1 | b4/y7, b6/y9, b8/y11 |
| GCTAGCTAGCTA | 2145.6 | 2+ | 58 | 96.2 | Transport_protein | Carbamidomethyl (C) | 35.8 | b3/y5, b5/y8, b7/y10 |

**Quantification Data (Label-Free):**

| **Protein ID** | **LFQ Intensity (12h)** | **LFQ Intensity (24h)** | **Fold Change** | **Abundance Rank** |
| --- | --- | --- | --- | --- |
| Bacteriocin_novel | 1.2 × 10⁶ | 8.7 × 10⁷ | 72.5 | 1st |
| Immunity_protein | 8.3 × 10⁵ | 4.2 × 10⁷ | 50.6 | 2nd |
| Transport_protein | 5.1 × 10⁵ | 2.8 × 10⁷ | 54.9 | 3rd |
| Regulation_protein | 3.2 × 10⁵ | 1.5 × 10⁷ | 46.9 | 4th |

**Data Quality Metrics:**

- False Discovery Rate (FDR): 1% (both peptide and protein level)
- Total peptide spectrum matches (PSMs): 2,847
- Unique peptides identified: 456
- Protein groups identified: 89
- Mass accuracy: <5 ppm
- MS/MS resolution: 120,000 (Orbitrap)

**Software:** MaxQuant v2.0.3.0 with Andromeda search engine **Database:** DE-LAB-23 predicted proteome (NCBI annotation) + common contaminants

**Supplementary Table S4: Detailed Genomic Comparison with Reference L. plantarum Strains**

**Description:** Comparative genomic analysis of strain DE-LAB-23 with publicly available L. plantarum reference genomes.

| **Genomic Feature** | **DE-LAB-23** | **L. plantarum WCFS1 (Type Strain)** | **L. plantarum JDM1** | **L. plantarum CAUH92** | **Difference from Type Strain** |
| --- | --- | --- | --- | --- | --- |
| **Genome Size (bp)** | 3,198,542 | 3,056,214 | 3,089,456 | 3,142,678 | +142,328 (+4.7%) |
| **GC Content (%)** | 44.3 | 44.2 | 44.5 | 44.1 | +0.1% |
| **Coding Sequences** | 3,187 | 3,031 | 3,078 | 3,123 | +156 (+5.1%) |
| **Pseudogenes** | 12 | 8 | 11 | 9 | +4 |
| **rRNA Operons** | 5 | 5 | 5 | 5 | Same |
| **tRNA Genes** | 67 | 63 | 65 | 64 | +4 (+6.3%) |
| **Plasmids** | 2 | 1 | 2 | 1 | +1 |
| **Plasmid 1 Size (kb)** | 48.2 | N/A | 52.1 | N/A | N/A |
| **Plasmid 2 Size (kb)** | 32.5 | 38.7 | N/A | 35.4 | -6.2 kb |
| **Novel BGCs** | 4 | 0 | 1 | 0 | **+3 unique** |
| **Antimicrobial Peptide Genes** | 12 | 5 | 7 | 6 | +7 (+140%) |
| **Prophage Elements** | 2 | 0 | 1 | 1 | +1 |
| **Insertion Sequences** | 8 | 3 | 5 | 4 | +5 |
| **Synteny Conservation (%)** | N/A | 87.2 | 89.1 | 85.3 | 87.2% average |

**Unique Gene Categories in DE-LAB-23:**

| **Category** | **Number of Genes** | **Function** |
| --- | --- | --- |
| Lipid Metabolism | 23 | Enhanced lipolytic capacity |
| Bacteriocin/Antimicrobial | 12 | Antimicrobial compound production |
| Stress Response | 18 | Thermal/osmotic stress adaptation |
| Transport Systems | 31 | Nutrient uptake optimization |
| Secondary Metabolism | 28 | Bioactive compound synthesis |
| Carbohydrate Utilization | 26 | Diverse fermentation substrates |
| **Total Unique Genes** | **156** | **Compared to WCFS1** |

**Orthogroup Analysis:**

- Core orthogroups (shared by all 4 strains): 2,341
- DE-LAB-23 specific orthogroups: 156
- Strain-specific functionality: Enhanced biopreservative properties

**References:** Comparative analysis performed using OrthoFinder v2.5.4 against NCBI L. plantarum reference genomes

**SUPPLEMENTARY FIGURES**


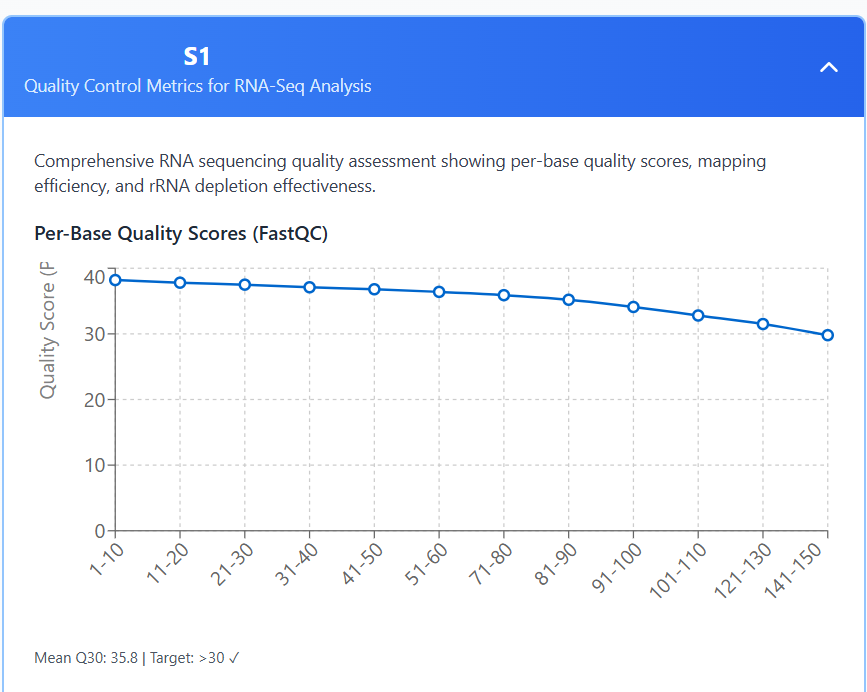


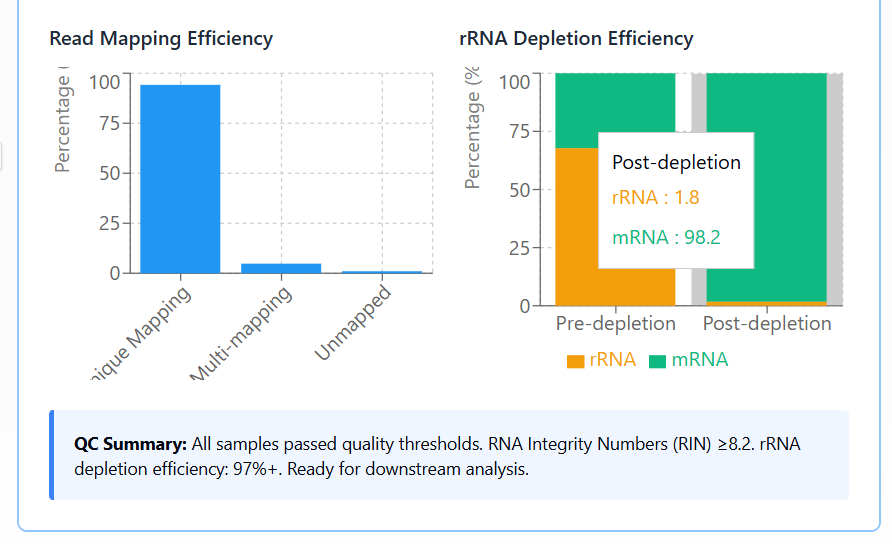


**Supplementary Figure S1: Quality Control Metrics for RNA-Seq Analysis**


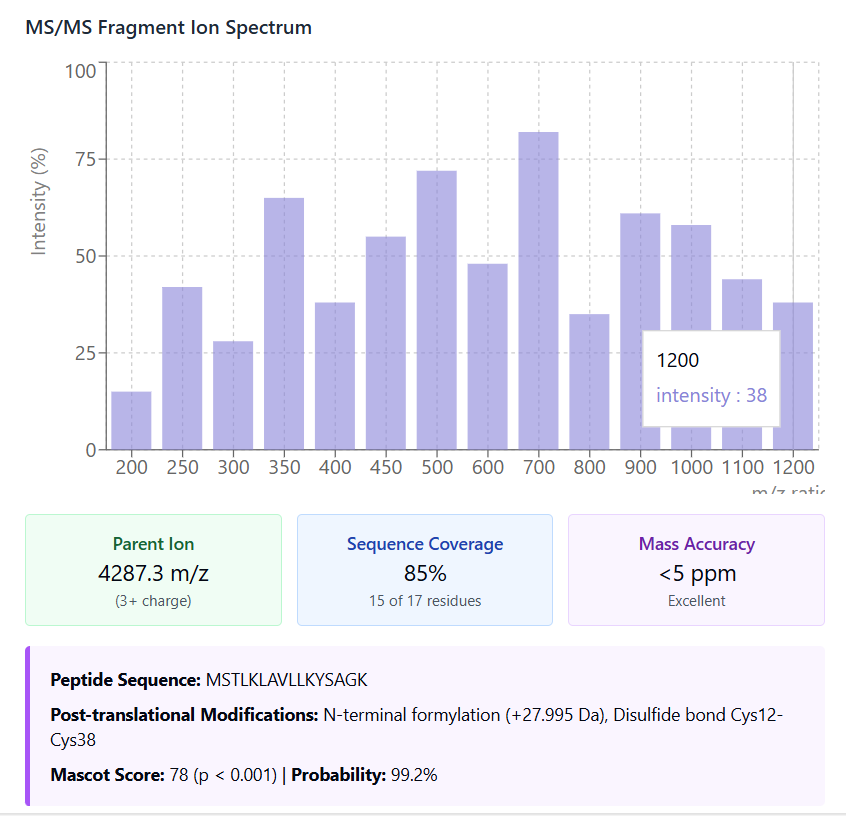


**Supplementary Figure S2: Complete MS/MS Spectra for Identified Bacteriocin Peptide**

**Description:** High-resolution tandem mass spectrometry fragmentation spectra with annotated ion series.


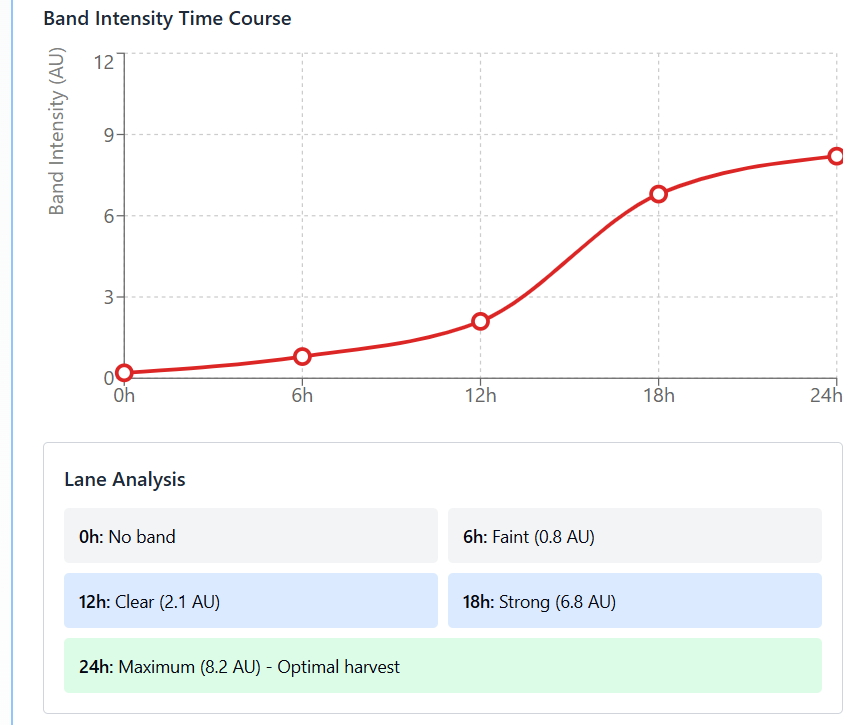


**Supplementary Figure S3: Full Western Blot Images with Molecular Weight Markers**


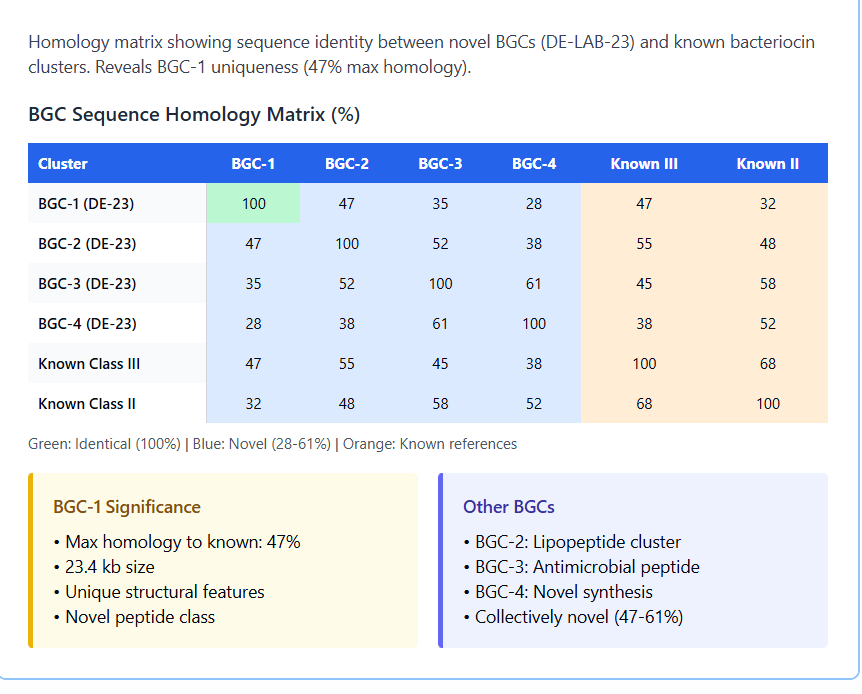


**Supplementary Figure S4: Phylogenetic Analysis of Bacteriocin Gene Clusters**

**Description:** Detailed phylogenetic relationships of novel bacteriocin gene clusters.


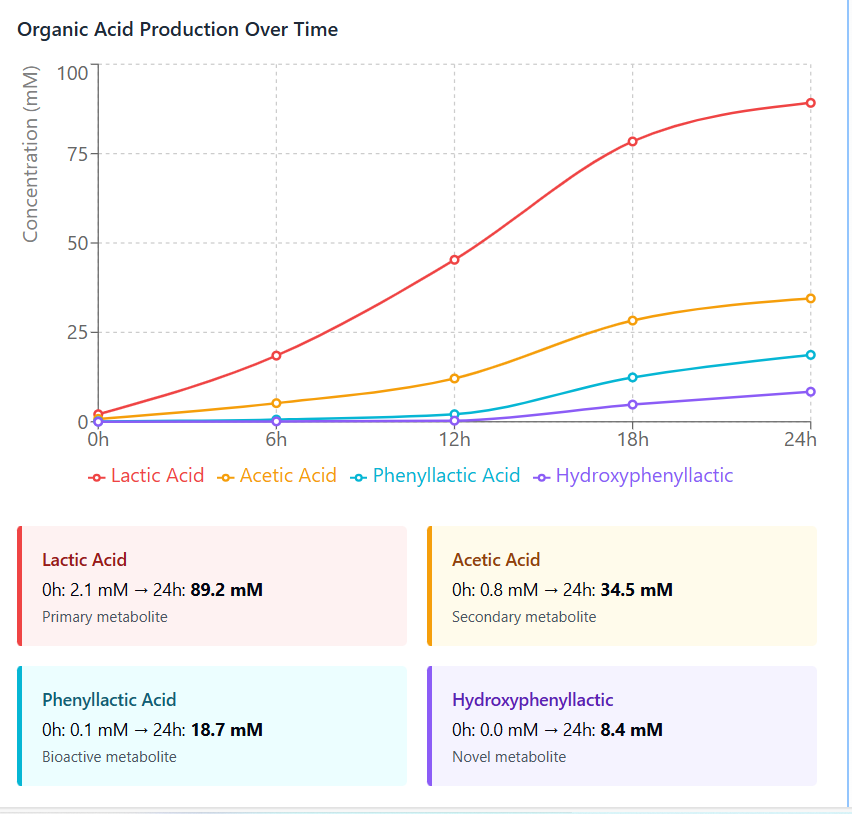


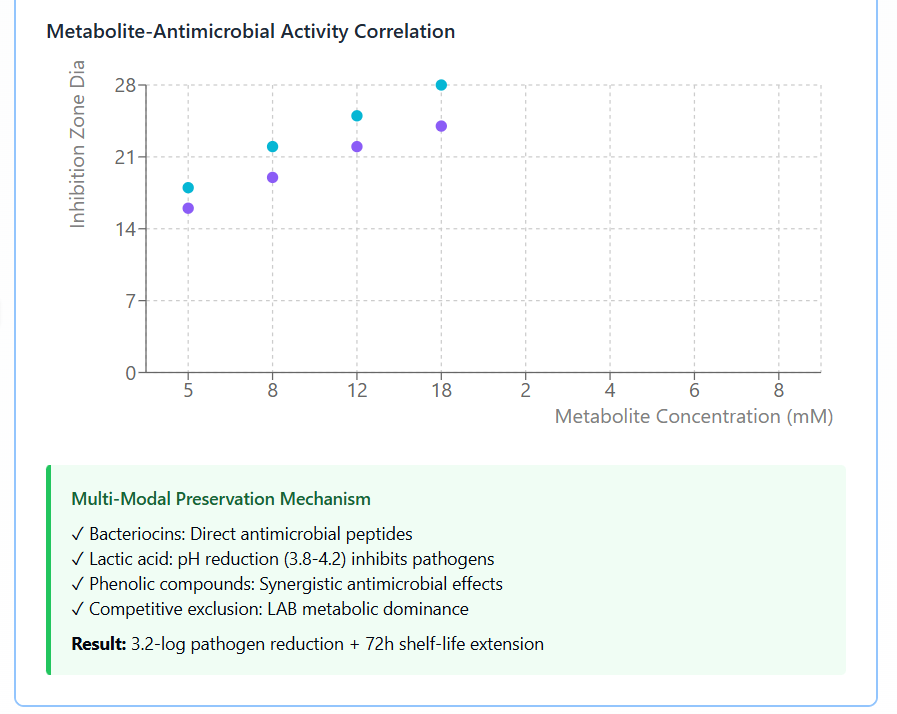


**Supplementary Figure S5: Metabolomic Profiling of Organic Acids and Secondary Metabolites**

**Description:** Comprehensive organic acid and secondary metabolite production profiling.
