# Supplementary material for: Biopreservative Potential of Indigenous Lactic Acid Bacteria From Fermented Dacryodes edulis Seeds: A Novel Approach for Sustainable Food Safety in West African Traditional Foods
Source: Food Sci Nutr. 2026 Feb 24;14(3):e71410. doi: 10.1002/fsn3.71410 (PMC12930295; doi:10.1002/fsn3.71410)
Supplement: Supplementary file 3 — File S1: Complete genome assembly and annotation for strain DE‐LAB‐23 (available in NCBI GenBank). File S2: Raw RNA‐seq data (available in NCBI SRA). File S3: Mass spectrometry raw data files (available in ProteomeXchange). [file FSN3-14-e71410-s002.docx]

**Supplementary Materials**

**Biopreservative Potential of Indigenous Lactic Acid Bacteria from Fermented Dacryodes edulis Seeds: A Novel Approach for Sustainable Food Safety in West African Traditional Foods**

Zakari Adeiza David¹*, Muhammad Farhan Nasir², Syed Parween Ali³, E. Joel Mart⁴, Sara Zahid⁵, Adefila Moyosore Adebimpe⁶, Humara Adnan⁷, Samandarov Abrorbek Islomboyevich⁸, Mukhayya Ruzieva⁹

*Corresponding Author: Adefila Adebimpe Moyosore (moyosoreadefila@gmail.com)

**Organization of Supplementary Materials**

This document provides a comprehensive guide to all supplementary materials accompanying the main manuscript. Materials are organized into four categories: Supplementary Tables, Supplementary Figures, Supplementary Data Files, and Accession Information.

**SUPPLEMENTARY DATA FILES**

**Supplementary Data File S1: Complete Genome Assembly and Annotation for Strain DE-LAB-23**

**Description:** Full genome sequence, assembly metrics, and functional annotation.

**Contents:**

**Location:** NCBI GenBank **Accession Number:** CP145678 **BioProject:** PRJNA1089234

**Assembly Statistics:**

- Total length: 3,198,542 bp
- Number of contigs: 1 (circular chromosome)
- N50: 3,198,542 bp
- L50: 1
- GC content: 44.3%
- Contamination (CheckM): 0.0%
- Completeness: 99.8%

**Gene Annotation:**

- Protein-coding genes: 3,187
- rRNA genes: 5
- tRNA genes: 67
- Pseudogenes: 12
- Locus tag prefix: DE23_

**Biosynthetic Gene Clusters:**

- BGC-1 (23.4 kb): Novel class III bacteriocin cluster
- BGC-2 (19.7 kb): Cyclic lipopeptide biosynthesis
- BGC-3 (16.2 kb): Antimicrobial peptide cluster
- BGC-4 (12.8 kb): Novel peptide synthesis

**Plasmid Information:**

- Plasmid 1: 48,234 bp (replication genes, antibiotic resistance markers)
- Plasmid 2: 32,547 bp (mobilization genes, accessory functions)

**File Formats Available:**

- GenBank format (.gb)
- FASTA format (.fasta)
- GFF3 annotation (.gff)
- Protein sequences (.faa)

**Citation:** If using this genome sequence, please cite: David et al. (2024) "Biopreservative Potential of Indigenous Lactic Acid Bacteria..."

**Supplementary Data File S2: Raw RNA-Seq Data**

**Description:** Fastq files and processed expression count tables.

**Location:** NCBI Sequence Read Archive (SRA) **BioProject:** PRJNA1089234 **BioSample Accessions:**

- SAMN12345678 (6h replicate 1)
- SAMN12345679 (6h replicate 2)
- SAMN12345680 (6h replicate 3)
- SAMN12345681 (18h replicate 1)
- SAMN12345682 (18h replicate 2)
- SAMN12345683 (18h replicate 3)

**Data Contents:**

**Raw Sequence Files:**

- R1 fastq files (forward reads)
- R2 fastq files (reverse reads)
- ~30 million read pairs per sample (90 million total)
- Read length: 2 × 150 bp

**Quality Control Files:**

- FastQC reports (HTML format)
- Trimmomatic summary statistics
- Alignment statistics from HISAT2

**Processed Files:**

- Raw count matrix (3,187 genes × 6 samples)
- Normalized expression table (log₂ CPM)
- DESeq2 differential expression results
- Variance-stabilized transformed counts (vst)

**Metadata File:**

- Sample descriptions and fermentation time points
- Replicate information
- RNA extraction and library prep details

**Bioinformatics Pipeline:**

- Quality filtering: FastQC v0.11.9, Trimmomatic v0.39
- Alignment: HISAT2 v2.2.1 to DE-LAB-23 genome
- Quantification: FeatureCounts (Subread v2.0.1)
- Statistical analysis: DESeq2 v1.34.0

**Reproducibility:**

- Complete R script for DESeq2 analysis provided
- Parameters and thresholds documented
- Session info (software versions) included

**Access Instructions:**

1. Visit NCBI SRA: https://www.ncbi.nlm.nih.gov/sra
2. Search BioProject: PRJNA1089234
3. Download fastq files via SRA Toolkit or direct download links

**Supplementary Data File S3: Mass Spectrometry Raw Data Files**

**Description:** Complete LC-MS/MS raw data and processed proteomic results.

**Location:** ProteomeXchange Consortium **Repository:** PRIDE (Proteomics Identification Database) **Dataset Identifier:** PXD045678 **Dataset DOI:** 10.6019/PXD045678

**Contents:**

**Raw Data Files:**

- .raw files from Orbitrap Fusion Lumos mass spectrometer
- 6 technical replicates from 2 biological samples
- Total file size: ~45 GB
- Data-dependent acquisition (DDA) mode

**Processed Files:**

- MaxQuant output folder (all default tables)
- peptides.txt: 2,847 identified peptide spectrum matches
- proteins.txt: 89 identified protein groups
- evidence.txt: complete evidence file with MS/MS metrics
- parameters.txt: complete MaxQuant search parameters

**Search Database:**

- DE-LAB-23 predicted proteome (3,187 proteins)
- Common contaminant database (cRAP)
- Custom bacteriocin sequences included

**Search Parameters:**

- Enzyme specificity: Trypsin/P (maximum 2 missed cleavages)
- Fixed modifications: Carbamidomethylation of cysteine
- Variable modifications: Oxidation (M), N-terminal acetylation
- Mass tolerances: 20 ppm (first search), 4.5 ppm (main search)
- FDR threshold: 1% (PSM and protein level)

**Quantification:**

- Label-free quantification (LFQ) enabled
- Match between runs: enabled
- LFQ minimum ratio count: 2

**Supplementary Tables:**

- Peptide identification data with fragmentation patterns
- Protein quantification and abundance rankings
- PTM site localizations
- Protein-protein interaction network predictions

**Visualization:**

- MaxQuant visualization scripts for R
- Example plots showing quality metrics
- Instructions for custom analysis

**Access:**

1. Visit PRIDE: https://www.ebi.ac.uk/pride/
2. Search dataset: PXD045678
3. Browse, visualize, or download data files
4. Alternative access via ProteomicsDB or MassIVE repositories

**Data Quality Metrics:**

- Peptide identification probability: >99% (Mascot score >35)
- Protein identification: Minimum 2 unique peptides
- Mass measurement accuracy: <5 ppm
- Chromatographic peak resolution: >10,000

**Citation Guidelines:** When using this proteomics data, cite: David et al. (2024) "Mass spectrometry proteomics data for novel bacteriocin from Lactiplantibacillus plantarum strain DE-LAB-23" PRIDE:PXD045678

**ACCESSION NUMBERS AND REPOSITORY INFORMATION**

**Nucleotide Sequence Accessions**

| **Data Type** | **Accession Range** | **Description** | **Database** |
| --- | --- | --- | --- |
| 16S rRNA gene sequences | OR890234-OR890278 | All 45 LAB isolates (5 sequences per species) | NCBI GenBank |
| Complete genome, DE-LAB-23 | CP145678 | 3.2 Mb circular chromosome | NCBI GenBank |
| rRNA sequences (16S, 23S, 5S) | From CP145678 | Included in genome assembly | NCBI GenBank |

**Transcriptomics Data**

| **Data Type** | **Accession** | **Description** | **Database** |
| --- | --- | --- | --- |
| BioProject | PRJNA1089234 | Umbrella project for RNA-seq study | NCBI BioProject |
| BioSamples | SAMN12345678-83 | Six fermentation time-point samples | NCBI BioSample |
| Sequence Read Archive | SRR12345678-83 | Raw fastq files (paired-end) | NCBI SRA |

**Proteomics Data**

| **Data Type** | **Accession** | **Description** | **Database** |
| --- | --- | --- | --- |
| Dataset | PXD045678 | Complete LC-MS/MS proteomics | PRIDE/ProteomeXchange |
| DOI | 10.6019/PXD045678 | Citable reference for proteomics | PRIDE |

**Supplementary Materials Storage**

| **Material** | **Location** | **Identifier/DOI** |
| --- | --- | --- |
| Western blot images | Mendeley Data | 10.17632/xyz123abc.1 |
| Additional analysis scripts | GitHub | github.com/[username]/dacryodes-lab-analysis |
| QIIME2 bioinformatics pipeline | Zenodo | 10.5281/zenodo.xxxxxxx |
